# Microbial Nitrogen Metabolism in Chloraminated Drinking Water Reservoirs

**DOI:** 10.1128/mSphere.00274-20

**Published:** 2020-04-29

**Authors:** Sarah C. Potgieter, Zihan Dai, Stephanus N. Venter, Makhosazana Sigudu, Ameet J. Pinto

**Affiliations:** a Department of Biochemistry, Genetics and Microbiology, University of Pretoria, Pretoria, South Africa; b Department of Civil and Environmental Engineering, Northeastern University, Boston, Massachusetts, USA; c Infrastructure and Environment Division, School of Engineering, University of Glasgow, Glasgow, United Kingdom; d Scientific Services, Rand Water, Vereeniging, South Africa; University of Wisconsin—Madison

**Keywords:** chloramination, drinking water systems, metagenomics, nitrification, nitrogen metabolism

## Abstract

Ammonia availability due to chloramination can promote the growth of nitrifying organisms, which can deplete chloramine residuals and result in operational problems for drinking water utilities. In this study, we used a metagenomic approach to determine the identity and functional potential of microorganisms involved in nitrogen biotransformation within chloraminated drinking water reservoirs. Spatial changes in the nitrogen species included an increase in nitrate concentrations accompanied by a decrease in ammonium concentrations with increasing distance from the site of chloramination. This nitrifying activity was likely driven by canonical ammonia-oxidizing bacteria (i.e., *Nitrosomonas*) and nitrite-oxidizing bacteria (i.e., *Nitrospira*) as well as by complete-ammonia-oxidizing (i.e., comammox) *Nitrospira*-like bacteria. Functional annotation was used to evaluate genes associated with nitrogen metabolism, and the community gene catalogue contained mostly genes involved in nitrification, nitrate and nitrite reduction, and nitric oxide reduction. Furthermore, we assembled 47 high-quality metagenome-assembled genomes (MAGs) representing a highly diverse assemblage of bacteria. Of these, five MAGs showed high coverage across all samples, which included two *Nitrosomonas, Nitrospira, Sphingomonas*, and *Rhizobiales*-like MAGs. Systematic genome-level analyses of these MAGs in relation to nitrogen metabolism suggest that under ammonia-limited conditions, nitrate may be also reduced back to ammonia for assimilation. Alternatively, nitrate may be reduced to nitric oxide and may potentially play a role in regulating biofilm formation. Overall, this study provides insight into the microbial communities and their nitrogen metabolism and, together with the water chemistry data, improves our understanding of nitrogen biotransformation in chloraminated drinking water distribution systems.

**IMPORTANCE** Chloramines are often used as a secondary disinfectant when free chlorine residuals are difficult to maintain. However, chloramination is often associated with the undesirable effect of nitrification, which results in operational problems for many drinking water utilities. The introduction of ammonia during chloramination provides a potential source of nitrogen either through the addition of excess ammonia or through chloramine decay. This promotes the growth of nitrifying microorganisms and provides a nitrogen source (i.e., nitrate) for the growth for other organisms. While the roles of canonical ammonia-oxidizing and nitrite-oxidizing bacteria in chloraminated drinking water systems have been extensively investigated, those studies have largely adopted a targeted gene-centered approach. Further, little is known about the potential long-term cooccurrence of complete-ammonia-oxidizing (i.e., comammox) bacteria and the potential metabolic synergies of nitrifying organisms with their heterotrophic counterparts that are capable of denitrification and nitrogen assimilation. This study leveraged data obtained for genome-resolved metagenomics over a time series to show that while nitrifying bacteria are dominant and likely to play a major role in nitrification, their cooccurrence with heterotrophic organisms suggests that nitric oxide production and nitrate reduction to ammonia may also occur in chloraminated drinking water systems.

## Abstract (precis)

Chloramines are often used as a secondary disinfectant when free chlorine residuals are difficult to maintain. However, chloramination is often associated with the undesirable effect of nitrification, which results in operational problems for many drinking water utilities. The introduction of ammonia during chloramination provides a potential source of nitrogen either through the addition of excess ammonia or through chloramine decay. This promotes the growth of nitrifying microorganisms and provides a nitrogen source (i.e., nitrate) for the growth for other organisms. While the roles of canonical ammonia-oxidizing and nitrite-oxidizing bacteria in chloraminated drinking water systems have been extensively investigated, those studies have largely adopted a targeted gene-centered approach. Further, little is known about the potential long-term cooccurrence of complete-ammonia-oxidizing (i.e., comammox) bacteria and the potential metabolic synergies of nitrifying organisms with their heterotrophic counterparts that are capable of denitrification and nitrogen assimilation. This study leveraged data obtained for genome-resolved metagenomics over a time series to show that while nitrifying bacteria are dominant and likely to play a major role in nitrification, their cooccurrence with heterotrophic organisms suggests that nitric oxide production and nitrate reduction to ammonia may also occur in chloraminated drinking water systems.

## INTRODUCTION

Disinfection of drinking water is often considered key in the management of microbial growth and the maintenance of water quality in most parts of the world, with the exception of some countries in Europe where microbial growth in disinfectant-residual-free drinking water distribution systems (DWDSs) is managed through nutrient limitation (1, 2). Chlorine is typically used as a primary disinfectant in most drinking water treatment plants (DWTP) and is often also used as the residual disinfectant during drinking water distribution. Challenges with respect to the stability of chlorine and the formation of disinfection by-products (3, 4) have prompted a shift toward the use of chloramines as the residual disinfectant during drinking water distribution. Chloramines show greater stability than free chlorine in the DWDS during transmission over long distances and increased efficiency in reducing biofilm growth and produce lower concentrations of regulated disinfection by-products (5–8).

However, in chloraminated systems, the introduction of ammonia provides an alternative source of nitrogen and growth substrate for ammonia-oxidizing microorganisms (AOM), through excess free ammonia and/or chloramine decay (7, 8). This promotes the growth of nitrifying bacteria and archaea, leading to nitrification (9, 10). Nitrification is an essential process in the biogeochemical nitrogen cycle and links the aerobic and anaerobic pathways of the nitrogen cycle by delivering nitrite and nitrate as electron acceptors for dissimilatory nitrate reduction, denitrification, respiratory ammonification, and anaerobic ammonia oxidation (11, 12). Traditionally, biologically mediated nitrification has been considered to be a two-organism process: first, ammonium (NH_4_^+^) is oxidized to nitrite (NO_2_-) by chemolithoautotrophic ammonia-oxidizing bacteria or ammonia-oxidizing archaea (AOB or AOA, respectively) (13, 14) followed by oxidation of nitrite to nitrate (NO_3_-) by chemolithoautotrophic nitrite-oxidizing bacteria (NOB) (15–17). NOB are often the principal biological source of nitrate, which not only is an important source of biologically available nitrogen for other microorganisms but also serves as an electron acceptor in the absence of oxygen. In contrast to canonical AOB/AOA and NOB, complete-ammonia-oxidizing (i.e., comammox) bacteria can completely oxidize ammonia to nitrate and are thus far believed to belong solely to the genus *Nitrospira* (phylum: *Nitrospirota*) (18–21). Bacterial nitrification in the DWDS causes depletion of chloramine residuals and disinfection decay. The resulting formation of nitrite in the system is also problematic as it can rapidly decrease levels of free chlorine and is also further oxidized, leading to an accelerated decrease in residual chloramine (15, 16). Further, due to its toxicity, the regulated concentration of nitrite is typically very low (1.0 mg/liter as N; U.S. EPA). While ammonia and nitrite can serve as an energy source for nitrifiers (22, 23), the loss of chloramine residuals can also lead to heterotrophic bacterial growth and biofilm accumulation, causing operational problems for many drinking water utilities (5, 22, 23).

In a previous study by Potgieter et al. (24) involving amplicon sequencing of the 16S rRNA gene, *Nitrosomonas* spp. were identified as dominant bacteria in the chloraminated sections of the DWDS, suggesting that nitrogen biotransformation led by ammonia oxidation and (potentially) nitrification may be important processes in this DWDS. The principal goal of the current study was to understand the functional potential of the microbial community involved in nitrogen biotransformation in a chloraminated DWDS (24). Using a genome-resolved metagenomic approach, the bacterial community and their genes involved in the nitrogen cycle were analyzed to develop a complete picture of the microorganisms and of the functional mechanisms likely to be involved in nitrogen biotransformation. The use of shotgun metagenomic sequencing allowed us to (i) avoid primer bias of gene-centered assays, (ii) assemble gene operons through *de novo* assembly, and (iii) to pinpoint functions to genome bins, which can provide a phylogenetic context for the nitrogen biotransformation potential. Therefore, using this approach, we aimed to explore the metabolic potential of nitrogen metabolism in chloraminated drinking water reservoirs by (i) investigating the taxonomic profile of the microbial community and the specific genes involved in nitrogen metabolism, (ii) identifying the processes that drive nitrogen transformation in chloraminated drinking water, and (iii) identifying the roles of the dominant microorganisms likely to play a role in nitrogen biotransformation by systematically analyzing their genomic content.

## RESULTS

### Spatial and temporal changes in ammonium, nitrite, and nitrate concentrations.

**FIG 1:**
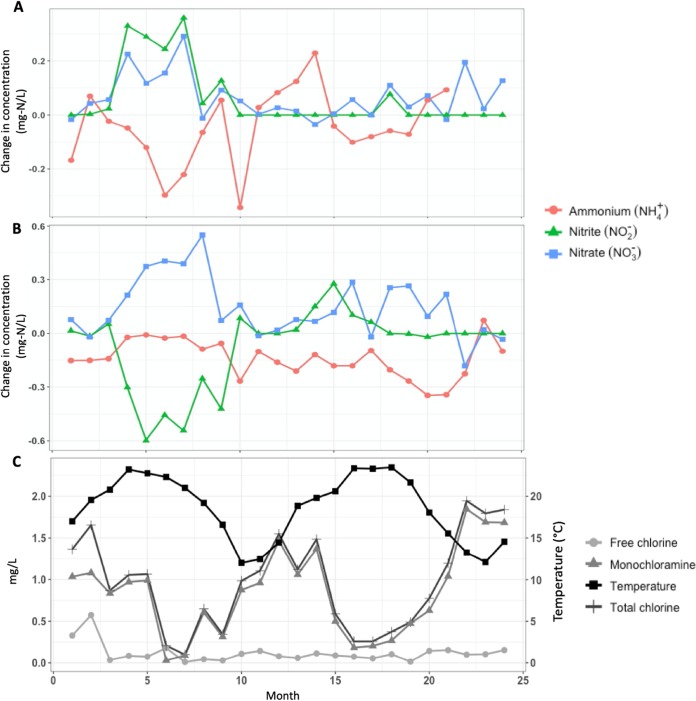
(A and B) Change in nitrogen species concentration (i.e., ammonium [red circles], nitrite [green triangles], and nitrate [blue squares]) (A) before and after passage through reservoir 1 (RES1) and (B) before and after passage through reservoir 2 (RES2) over the 2-year sampling period. (C) Average concentrations of disinfectant residuals across both reservoirs (i.e., free chlorine [circles], total chlorine [crosses], and monochloramine [triangles]) together with average temperature (black line with squares).

Variation in the concentrations of ammonium (NH_4_^+^), nitrite (NO_2_-), and nitrate (NO_3_-) indicated differing degrees of nitrification in both reservoir 1 (RES1) and RES2 (see Fig. S1 in the supplemental material). Typically, a negative correlation was observed between ammonia concentrations and both nitrite and nitrate concentrations (Fig. 1). In samples obtained at the outflows of both reservoirs, decreases in ammonium concentrations were associated with increases in nitrite and nitrate concentrations (Fig. 1A and B). Interestingly, a significant decrease in nitrite was concomitant with an increase in nitrate in the first year (months 4 to 9) in samples following RES2, suggesting complete nitrification (Fig. 1B). In addition, nitrification in RES1 and RES2 exhibited some temporal dynamics, differing specifically between the first year and second year of the study. The observed temporal trends in nitrification are likely associated with changes in temperature and disinfectant residual concentrations (Fig. 1C). Disinfection residual concentrations were typically higher in the winter and spring months and correlated negatively with water temperature (Fig. 1C). Conversely, nitrite and nitrate concentrations were highest in summer and autumn months where associated ammonium concentrations were low.

10.1128/mSphere.00274-20.1 FIG S1 Temporal changes in nitrogen compounds (i.e., ammonium [red circles], nitrite [green triangles], and nitrate [blue squares]) over 2 years. The plots in panels A and B present data determined before and after the water passed through RES1, respectively, and plots C and D present data determined before and after the water passed through RES2, respectively. Download FIG S1, PDF file, 0.2 MB. Copyright © 2020 Potgieter et al. 2020 Potgieter et al. This content is distributed under the terms of the Creative Commons Attribution 4.0 International license.

### Differences in microbial community composition between the two reservoirs.

The majority of small-subunit (SSU) rRNA gene sequences retrieved from the metagenomic assembly were identified as bacterial in origin (see Table S1 in the supplemental material). *Proteobacteria* was the most abundant phylum in both reservoirs, followed by *Nitrospirota*. The relative abundances of *Alphaproteobacteria* and *Gammaproteobacteria* (specifically order: *Betaproteobacteriales*) differed between RES1 and RES2 (Table S1). Within *Betaproteobacteriales*, SSU rRNA genes from *Nitrosomonas* were highly abundant (mean relative abundance [MRA] values: 31.44% ± 15.18% in RES1 and 10.10% ± 10.07% in RES2), with two coexisting *Nitrosomonas* SSU rRNA genes detected in both reservoirs. Similarly, the phylum *Nitrospirota* (genus: *Nitrospira*) was significantly more abundant in RES1 than in RES2 (Table S1). However, RES1 was dominated by a single *Nitrospira* SSU rRNA gene, which cooccurred with a second *Nitrospira* gene in RES2. The *Nitrosomonas* and *Nitrospira* populations in RES1 also cooccurred with heterotrophic bacteria classified as *Rhizobiales*, *Sphingomonas*, *Methylobacterium*, *Hyphomicrobium*, and *Sideroxydans*. In contrast, RES2, which had significantly lower abundances of nitrifying organisms, was dominated by populations within the phyla *Gemmatimonadetes* (genus: *Gemmatimonas*), *Planctomycetes* (family: *Pirellulaceae*), and proteobacterial genera *Bosea* and *Hydrogenophaga*. These metagenomic-based SSU rRNA gene results are consistent with amplicon (i.e., 16S rRNA gene) sequencing results reported previously by Potgieter et al. (24). Differences in the microbial communities of the two reservoirs were also reflected in beta diversity measures (i.e., structure-based [Bray-Curtis] and membership-based [Jaccard] measures) where the microbial communities were on average 60% dissimilar in community structure between the two reservoirs (Bray-Curtis, 0.59% ± 0.16) and 74% dissimilar in community membership (Jaccard, 0.74 ± 0.12) (analysis of molecular variance [AMOVA], *F_ST_* ≤ 2.12, *P* < 0.05).

10.1128/mSphere.00274-20.3 TABLE S1 Percent coverage of SSU rRNA contigs identified as bacterial phyla, eukaryota, and unclassified taxa averaged across the two reservoirs (coverage was calculated for contigs >250 bp in length). Download Table S1, DOCX file, 0.01 MB. Copyright © 2020 Potgieter et al. 2020 Potgieter et al. This content is distributed under the terms of the Creative Commons Attribution 4.0 International license.

### Nitrogen biotransformation genes and their host associations.

**FIG 2:**
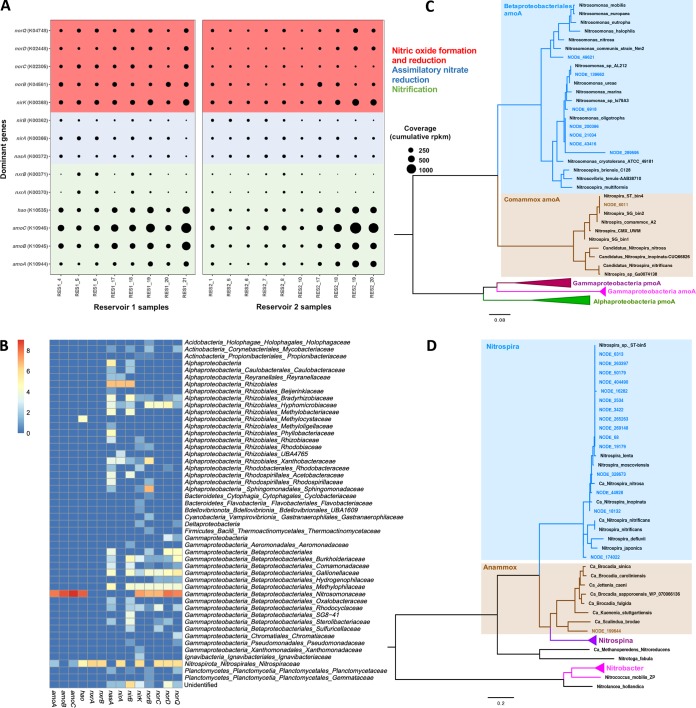
(A) Cumulative abundances (in reads per kilobase per million [RPKM]) of the dominant genes identified to be involved in the nitrogen cycle (i.e., genes with a cumulative abundance of >100 RPKM in both reservoirs) across all reservoir samples. (B) Family-level taxonomic distribution of nitrogen-transforming genes based on BLAST analyses. The heat map indicates presence/absence and relative abundance of dominant nitrogen biotransformation genes. (C and D) Phylogenetic placement of ammonia monooxygenase, subunit A (*amoA*) (C), and nitrite oxidoreductase, subunit A (*nxrA*) (D). Both maximum likelihood phylogenetic trees were constructed based on amino acid sequences from contigs identified as containing the respective genes. Genes identified from this study are colored according to cluster, while reference genes are in black.

A suite of nitrogen metabolism-related genes were identified in the metagenomes from both reservoirs using KEGG-based annotation. However, the level of coverage of many of these genes was low and, as a result, their contribution to overall nitrogen metabolism is likely to be limited. Therefore, we focus here only on genes with a cumulative abundance of greater than 100 reads per kilobase per million (RPKM) in both reservoirs. These genes include those involved in ammonia oxidation (i.e., *amoCAB* and *hao*), assimilatory nitrate reduction (*nasA*) and nitrite reduction (*nirA* and *nirB*), nitric oxide (NO)-forming nitrite reduction (*nirK*), nitric oxide reduction (*norCBQD*), and nitrite oxidation (*nxrAB*) (Table S2) (Fig. 2).

10.1128/mSphere.00274-20.4 TABLE S2 The cumulative abundance (expressed as reads per kilobase per million [RPKM]) of the dominant nitrogen-transforming genes (i.e., genes with cumulative coverage of >100 RPKM) in the two reservoirs. Download Table S2, DOCX file, 0.01 MB. Copyright © 2020 Potgieter et al. 2020 Potgieter et al. This content is distributed under the terms of the Creative Commons Attribution 4.0 International license.

While genes associated with nitrification were among the most abundant nitrogen metabolism genes (Fig. 2A; see also Table S2), they were primarily associated with canonical AOB (genus: *Nitrosomonas*), with one copy each of low-abundance *amoA*, *amoC*, *and hao* genes annotated as comammox *Nitrospira* bacteria based on their phylogenetic placement. BLAST results confirmed that the majority of *amoCAB* and *hao* genes represented members of *Nitrosomonas* genus (Fig. 2B). This was confirmed by demonstration of the phylogenetic placement of *amoA* genes, where nearly all *amoA* genes clustered with *Nitrosomonas* species, indicating limited diversity, and by findings indicating that the majority of *amoA* genes were associated with strict ammonia oxidizers (Fig. 2C). Interestingly, a single *amoA* gene grouped within the *Nitrospira* comammox cluster (Fig. 2C). BLAST results confirmed that this *amoA* contig represented a *Nitrospira* species, indicating the potential presence of a comammox bacterium within the microbial community. Nitrite oxidation genes encoding nitrite oxidoreductase (*nxrAB*) were also observed across all samples (Fig. 2A). The low coverage of individual *nxrAB* genes compared to *amoCAB* and *hao* genes suggests that while comammox bacteria (which contain genes for both ammonia and nitrite oxidation) were detected in the system, their contribution to the nitrification is likely to be minor compared to that of canonical AOB (Fig. 2A and B; see also Table S2). As expected, results of BLAST analyses of *nxrAB* genes confirmed that the majority of these genes represented *Nitrospira* species with low levels of diversity among nitrite-oxidizing bacteria (Fig. 2D). Also consistent with BLAST results, phylogenetic analyses confirmed that the majority *of* the *nxrA* genes belonged to widely distributed lineage II of the genus *Nitrospira* clustering with the canonical nitrite oxidizers N. lenta and N. moscoviensis and “*Candidatus* Nitrospira sp. ST-bin5” described by Wang et al. (25) (Fig. 2D). In addition, some *nxrA* genes grouped closely with known comammox *Nitrospira* species, including “*Candidatus* Nitrospira nitrosa,” “*Ca*. Nitrospira inopinata,” and “*Ca*. Nitrospira nitrificans.” However, identification and taxonomic resolution of comammox bacteria based on *nxrA* gene phylogeny are not feasible. Although different in the two reservoirs, the temporal trends in the cumulative coverage generally indicated a contrasting relationship between *amoCAB* and *nxrAB* genes. In RES1, *nxrAB* genes showed increased coverage in March (2015 and 2016) relative to *amoA* genes. Conversely, during months where *nxrAB* gene coverage decreased (April 2015 and 2016), the coverage of *amoCAB* genes increased. These contrasting temporal trends were also observed in RES2.

The higher abundance of nitrifying organisms was also associated with increased abundance of genes involved in nitric oxide formation via nitrite reduction (*nirK*) and nitric oxide reduction (*norCBQD*) to nitrous oxide. Here, a relatively high number of genes were identified as *nirK*, indicating high diversity associated within this function (Fig. 2B). This was confirmed by BLAST results where *nirK* genes were identified as belonging to *Nitrospira* (36%), *Alphaproteobacteria* (9%), and *Betaproteobacteriales* (34%), 21% of which were identified as *Nitrosomonadaceae*. BLAST analyses of individual *nor* genes revealed that they predominantly represented members of families within *Alphaproteobacteria* and *Betaproteobacteriales*. More specifically, *norB* genes represented families that included *Nitrosomonadaceae* (33%) and *Sphingomonaceae* (23%). The majority of *norQ* genes were identified as *Nitrospira* (16%) and *Nitrosomonadaceae* (40%) genes, 14% of which were identified as belonging to *Nitrosomonas* species. Similarly, the majority of *norC* genes were identified as *Nitrospira* (44%) and *Nitrosomonadaceae* (36%) genes. Lastly, as observed with the *norQ* and *norC* genes, 55% of the *norD* genes were found to represent members of *Nitrosomonadaceae* and 13% to represent members of the genus *Nitrospira*. While nitrifiers, in particular, those of *Nitrospira* and *Nitrosomonas*, largely contributed to the prevalence of these genes, a large proportion were also annotated as originating from heterotrophs. For instance, another dominant source of annotated *nirK* genes was from the alphaproteobacterial order *Rhizobiales*, while the *nor* genes originated from gammaproteobacterial families of *Methylophilaceae* and *Gallionellaceae* and alphaproteobacterial families of *Hyphomicrobiaceae* and *Sphingomonadaceae*. These observed cooccurrences of nitrogen metabolism genes of nitrifiers and heterotrophs are consistent with the 16S rRNA gene results. Interestingly, the temporal cumulative coverage of *norB* generally showed a converse relationship to the cumulative coverage of other *nor* genes, specifically, *norC* and *norQ.* The observed level of diversity among *nor* genes was high, and their dynamics may be associated with different members of the community, resulting in differences in their levels of coverage (Fig. 2B).

Functional potential associated with assimilation of nitrite and nitrate exhibited higher redundancy and taxonomic diversity. For instance, the cytoplasmic assimilatory nitrate reductase *nasA* gene (encoding the catalytic subunit of the NADH-nitrate reductase) was identified as the most abundant nitrate reductase gene, with a high cumulative level of coverage (>100 RPKM) across both RES1 and RES2 (Fig. 2B; see also Table S2). Dissimilatory nitrate reductase genes were also identified, including respiratory membrane-bound nitrate reductase genes (*narGHJI*), although their cumulative level of coverage of was low (<100 cumulative RPKM in both reservoirs). BLAST analyses revealed that a diverse assemblage of bacterial community members had the potential to use nitrate as an alternative electron acceptor. The majority of *nasA* genes were represented by members of *Alphaproteobacteria* (66% of *nasA* genes), among which 34% were identified as belonging to the order *Rhizobiales*, including predominantly the families *Bradyrhizobiaceae*, *Rhodospirillaceae*, and *Hyphomicrobiaceae.* In addition, *Betaproteobacteriales* represented another 18% of the *nasA* genes (Fig. 2B). Genes encoding enzymes involved in the assimilatory reduction of nitrite to ammonia, i.e., ferredoxin-nitrite reductase (*nirA* gene) and nitrate reductase (NADH), large subunit (*nirB* gene), also showed high cumulative levels of coverage (i.e., cumulative coverage of >100 RPKM) in RES1 and RES2; however, the abundance of *nirB* genes was less than that of the *nirA* genes (Fig. 2B; see also Table S2). Although both the *nirA* and *nirB* genes showed moderate to low coverage across both reservoirs, the number of genes identified as belonging to these functions, specifically, *nirB* genes, was high. BLAST analyses revealed that 50% of the *nirA* genes represented members of *Alphaproteobacteria*, 36% of which were identified as *Rhizobiales*, including the families *Bradyrhizobiaceae*, *Hyphomicrobiaceae*, and *Methylobacteriaceae*. In addition, 32% of *nirA* genes were identified as belonging to *Nitrospira* spp. The majority of *nirB* genes represented *Betaproteobacteriales* (69% of *nirB* genes) and were predominately made up of members of the families *Gallionellaceae*, *Burkholderiaceae*, and *Comamonadaceae.* In addition, another 21% of the *nirB* genes were found to represent *Alphaproteobacteria* (order: *Rhizobiales*), including mostly the families *Bradyrhizobiaceae* and *Methylobacteriaceae.* This diversity among *nirA* and *nirB* genes was also reflected in their presence and/or distribution throughout the 47 MAGs (see below). Temporal trends in the relative abundances of the *nasA* and *nirA* genes were observed to have a converse relationship in RES1. Increases in the relative abundance of *nirA* genes (in February and March 2015 and March 2016) were associated with a decreased relative abundance of *nasA* genes. However, this converse relationship was not observed between *nasA* and *nirB* in RES2. In RES2, the relative abundances of these genes (*nasA*, *nirA*, and *nirB*) generally showed the same temporal trends across RES2 samples and the same converse relationship with ammonia concentrations, specifically between *nasA* and *nirA*. Increases in the relative abundances of both *nasA* and *nirA* genes were observed in April and May 2015 and March 2016. This suggests the potential for complete assimilatory nitrate reduction to ammonia (i.e., *nasA*, *nirA*, and *nirB*), specifically in RES2 (decreased ammonium concentrations) as the relative abundance of *nasA* genes typically showed a converse relationship with ammonia concentrations. In addition, temporal trends among *nirA* and *nirB* genes showed a converse relationship with *nirK* genes in both reservoirs.

### The nitrogen metabolic potential of dominant metagenome-assembled genomes (MAGs).

**FIG 3:**
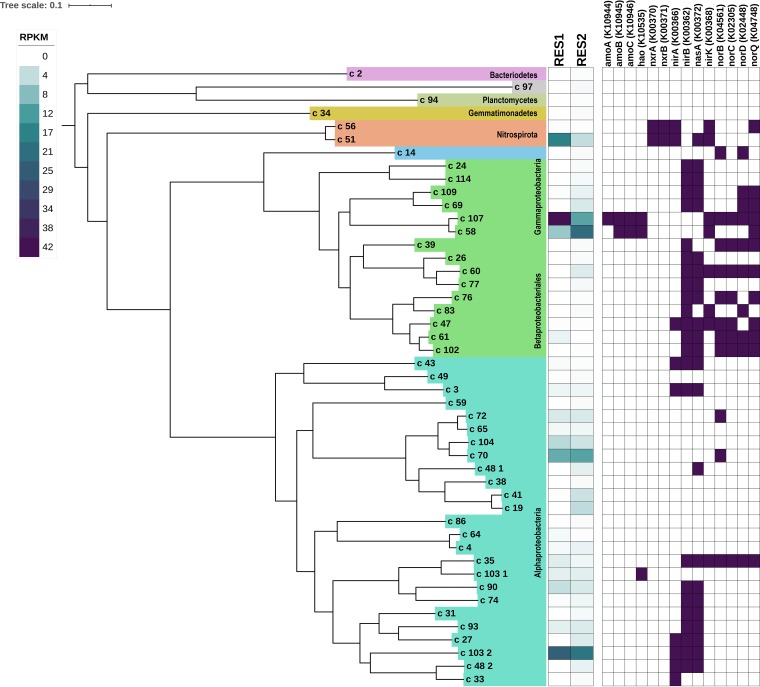
Phylogenomic tree for the 47 metagenome-assembled genomes (MAGs) obtained as part of this study. The phylogenomic tree was constructed using a concatenated alignment of 48 single-copy core genes. The average relative abundances (in reads per kilobase per million [RPKM]) of all MAGs in reservoir 1 (RES1) reservoir and reservoir 2 (RES2) are shown as a heat map, followed by data indicating of the presence or absence of the dominant genes involved in nitrogen biotransformation (i.e., genes with a cumulative abundance of >100 RPKM in both reservoirs) in all MAGs.

Following metagenomic binning, we obtained 47 high-quality constructed MAGs, which included members of *Alphaproteobacteria* (*n* = 25), *Betaproteobacteriales* (*n* = 15), *Nitrospirae* (*n* = 2), *Planctomycetes* (*n* = 2), *Bacteroidetes* (*n* = 1), *Gammaproteobacteria* (*n* = 1), and *Gemmatimonadetes* (*n* = 1) (Fig. 3A). A complete overview of the 47 MAGs is provided in Table S3. Of the 47 MAGs, 5 dominated the microbial community (i.e., cumulative coverage of >100 RPKM) across both reservoirs (Fig. 3). These MAGs were identified as two *Nitrosomonas*-like MAGs (C58 and C107), a *Rhizobiales*-like MAG (C103.2), a *Sphingomonas*-like MAG (C70), and a *Nitrospira*-like MAG (C51). The *Nitrosomonas*-like MAG (C107) exhibited the highest abundance in the metagenomic data set (Fig. 3). Phylogenetic analysis of the SSU rRNA gene containing contigs associated with the *Nitrosomonas*-like MAGs confirmed that they both clustered with N. oligotropha, with good bootstrap support (see Fig. S2A). This is consistent with members of N. oligotropha lineage being adapted to environments with low to moderate concentrations of ammonia. Although the two *Nitrosomonas*-like MAGs were potentially identified as the same species (based on SSU rRNAs), differences in the average levels of coverage of these MAGs across the two reservoirs suggested that they may exhibit competitive dynamics and may represent two separate populations of *Nitrosomonas*. Here, the coverage of *Nitrosomonas*-like MAG (C58) increased (although not significantly) from RES1 to RES2 and, conversely, that the coverage of *Nitrosomonas*-like MAG (C107) decreased significantly from RES1 to RES2 (*P* < 0.001) (Table S4). Spearman correlations revealed that there was a moderate negative correlation between these two MAGs in RES1; however, this was not statistically significant (Spearman correlation of −0.40, *P* = 0.3268). Within both of the *Nitrosomonas*-like MAGs, the majority of the genes required for ammonia oxidation were detected. The exception was a lack of an *amoA* gene in a *Nitrosomonas-*like MAG (C58), which may have been due to its overall lower abundance and thus to suboptimal assembly of its genome during the *de novo* assembly process. Both *Nitrosomonas*-like MAGs also contained *nirK* genes (nitric oxide-forming nitrite reductase genes) (Fig. 3). In addition, one *Nitrosomonas*-like MAG (C107) contained *norCBQD* genes, whereas another *Nitrosomonas*-like MAG (C58) contained only the *norQ* gene, suggesting that these dominant MAGs may play a role in regulating the concentrations of nitric oxide. Typically, *Nitrosomonas* spp. lack the *norCBQD* operon; however, acquisition of these genes in AOB genomes has been reported to occur through horizontal gene transfer (26). Spearman correlations between a *Nitrosomonas*-like MAG (C107) and other dominant MAGs revealed strong correlations specifically in RES2. Strong positive correlations were observed with a *Sphingomonas-*like MAG (C70) (Spearman correlation, 0.76; *P* < 0.05) and a *Rhizobiales*-like MAG (C103.2) (Spearman correlation, 0.72; *P* < 0.05). A *Nitrosomonas*-like MAG (C58) also showed a strong positive correlation with a *Rhizobiales*-like MAG (C103.2) (Spearman correlation, 0.81; *P* < 0.05).

10.1128/mSphere.00274-20.2 FIG S2 (A) Maximum likelihood phylogenetic tree showing the grouping of *Nitrosomonas* SSU rRNA contigs retrieved from metagenomic data with *Nitrosomonas* 16S rRNA reference strains with Methyloversatilis thermotolerans as the outgroup. (B) Maximum likelihood phylogenetic tree showing the grouping of *Nitrospira* SSU rRNA contigs retrieved from metagenomic data with *Nitrospira* 16S rRNA reference strains with Leptospirillum ferrooxidans as the outgroup. Both trees show bootstrap analysis of 1,000 replicates (bootstrap values are indicated as percentages). Download FIG S2, PDF file, 0.1 MB. Copyright © 2020 Potgieter et al. 2020 Potgieter et al. This content is distributed under the terms of the Creative Commons Attribution 4.0 International license.

10.1128/mSphere.00274-20.5 TABLE S3 Characteristics of constructed metagenome-assembled genomes (MAGs) and their coverage across both reservoirs. Download Table S3, DOCX file, 0.04 MB. Copyright © 2020 Potgieter et al. 2020 Potgieter et al. This content is distributed under the terms of the Creative Commons Attribution 4.0 International license.

10.1128/mSphere.00274-20.6 TABLE S4 Results from DESeq2 analyses comparing the number of reads mapping to each metagenome-assembled genome between the two reservoirs. Download Table S4, DOCX file, 0.02 MB. Copyright © 2020 Potgieter et al. 2020 Potgieter et al. This content is distributed under the terms of the Creative Commons Attribution 4.0 International license.

The *Rhizobiales*-like MAG (C103.2) showed high coverage and stable abundance across RES1 and RES2, constituting the second most abundant MAG across all samples (Fig. 3; see also Table S3). Its abundance showed a strong positive correlation with the most dominant nitrifier, i.e., *Nitrosomonas*-like MAG (C107). The nitrogen metabolism of this MAG included assimilatory nitrate and nitrite reduction (*nasA*, *nirA*, and *nirB*, respectively) (Fig. 3). BLAST analyses of the *nasA*, *nirA*, and *nirB* genes revealed a close relation to *Proteobacteria* bacterium ST_bin 15, assembled from tap water metagenomes, reported by Wang et al. (25). The taxonomic classification of this MAG, limited to the family level, could not be improved based on its SSU rRNA gene classification or through the use of genome-level taxonomy databases (e.g., GTDB-tk) (27). However, based on the phylogenomic inference, this MAG grouped closely with other MAGs within the family *Bradyrhizobiaceae*. This is consistent with a *Rhizobiales-*like MAG constructed by Chao et al. (28) from a drinking water biofilm exposed to disinfectant residuals (28). Furthermore, assessment of additional correlations between a *Rhizobiales*-like MAG (C103.2) and other dominant MAGs showed a strong positive correlation with a *Sphingomonas-*like MAG (C70) (Spearman correlation, 0.76; *P* < 0.05).

The same *Sphingomonas-*like MAG (C70) showed consistent coverage across both reservoirs, and its levels of abundance between reservoirs were not significantly different (*P* = 0.8) (Table S4). Its metabolic potential related to nitrogen biotransformation was largely limited to nitric oxide reductase (*norB*), which is involved in conversion of nitric oxide to nitrous oxide (Fig. 3). Further taxonomic classification of the SSU rRNA gene containing the contig and *norB* gene observed in this MAG confirmed its taxonomy as *Sphingomonas*. *Sphingomonas* spp. are common inhabitants of drinking water systems, and the dominance of this MAG within the community suggests that it may be involved in other important metabolic capabilities within the microbial community that extend beyond the nitrogen cycle, such as initial biofilm formation and subsequent maturation (29). *Sphingomonas* spp. are known to produce a wide variety of exopolymers (30, 31); among them, genes involved in the biosynthesis of sphingans, specifically, gellan (*rhsACBD* genes), were detected in this *Sphingomonas-*like MAG. Furthermore, KEGG module-based analyses suggests that the positive correlation observed between the *Sphingomonas-*like MAG and the *Nitrosomonas-*like MAGs may be linked to the ability of one MAG to produce and/or degrade certain amino acids and sugars where the other MAG cannot. Here, both *Nitrosomonas*-like MAGs (C58 and C107) had the potential for tyrosine biosynthesis whereas the *Sphingomonas-*like MAG had the potential for tyrosine degradation. The *Sphingomonas-*like MAG had the potential for methionine and nucleotide sugar (galactose to UDP-galactose) biosynthesis whereas the *Nitrosomonas*-like MAG did not. Conversely, the *Nitrosomonas*-like MAG had the potential for glycogen biosynthesis whereas the *Sphingomonas-*like MAG did not.

The *Nitrospira*-like MAG (C51) showed high coverage in RES2 (*P* < 0.001) (Fig. 3; see also Table S3). Phylogenetic analysis of SSU rRNA gene associated with this MAG showed close clustering with Nitrospira lenta (lineage II) (Fig. S2B). Investigations into the genetic potential of this MAG to transform nitrogen species confirmed that this *Nitrospira-*like MAG contained nitrite oxidoreductase genes (*nxrAB*). In addition, this MAG had the potential for other nitrogen-transforming reactions, as it contained genes involved in complete assimilatory nitrate reduction to ammonia (*nasA* and *nirA*, respectively) as well as the nitric oxide-forming nitrite reductase (*nirK*) (Fig. 3). Interestingly, while *amoA* genes associated with the comammox *Nitrospira* were identified within the metagenomes, this *Nitrospira*-MAG did not harbor any genes associated with ammonia oxidation (i.e., *amoA*, *amoB*, *amoC*, and *hao*), suggesting that it was a canonical lineage II nitrite-oxidizing bacteria (NOB). *Nitrospira*-like MAG (C51) had a strong negative correlation with *Nitrosomonas*-like MAG (C107) (Spearman correlation, −0.98; *P* < 0.001) in RES2. Interestingly, that *Nitrospira*-like MAG (C51) also showed a strong negative correlation with another *Nitrospira*-like MAG (C56) in RES1 (Spearman correlation, −0.79; *P* < 0.05) closely related to Nitrospira moscoviensis. That *Nitrospira*-like MAG (C56) was also determined to be a lineage II canonical NOB with very low abundance in both reservoirs for all time points. The abundance of the *Nitrospira*-like MAG (C51) was also negatively correlated with that of a *Sphingomonas-*like MAG (C70) (Spearman correlation, −0.73; *P* < 0.05) and a *Rhizobiales*-like MAG (C103.2) (Spearman correlation, −0.64; *P* < 0.05).

## DISCUSSION

### Differing stages of nitrification between the two reservoirs.

The water chemistry data indicated differing patterns in the concentrations of nitrogen species and spatial changes in disinfectant residuals between the two reservoirs. Differing stages of nitrification in different sections of a chloraminated DWDS had also been observed by Shaw et al. (32). Complete and partial nitrification levels were observed, specifically in RES1, as decreases in ammonium concentrations were associated with concomitant increases in nitrite and nitrate concentrations, particularly in the first year. While nitrification occurred in both reservoirs, it did not occur at all sampled time points. In RES1, this may have been a consequence of increased monochloramine concentrations, as months with increased monochloramine concentrations showed reduced nitrification rates. In addition, ammonium concentrations were consistently higher in RES1 than in RES2, as expected, as these samples were closer to the site of chloramination. Conversely, following RES2, samples (specifically those within the first year) had elevated nitrate concentrations with reduced nitrite and ammonium concentrations, suggesting that with increasing distance from the site of chloramination, samples nearing the end of the DWDS exhibited increasingly complete nitrification. Here, it was more likely that the depletion of nitrite might have been the result of tight coupling of canonical ammonia oxidation and nitrite oxidation (32, 33).

### Changes in bacterial community composition between the two reservoirs.

The majority of annotated proteins were bacterial in origin, and the taxonomic profiles determined on the basis of SSU rRNA genes were in agreement with previous descriptions of the microbial community within chloraminated DWDS, in which *Proteobacteria* and (to a lesser extent) *Nitrospira* were the dominant phyla (24, 34, 35). The change in dominance between *Alphaproteobacteria* and *Betaproteobacteriales* in RES1 and RES2 was also observed by Potgieter et al. (24). The variation in dominance between *Alphaproteobacteria* and *Betaproteobacteria* (now classified as the order *Betaproteobacteriales* in the phylum *Gammaproteobacteria*) has been well documented, where their dominance varies depending on multiple factors, including disinfectant residual concentrations (36, 37) and seasonal trends (38–41).

However, due to the lack of quantitative and viability assays, it is unclear what proportion of the community data represents viable or metabolically active cells. Although there is a lack of absolute abundance data in this study, it does not detract from the observed genetic potential of the microbial community to transform nitrogen. Further, the changes in abundance of nitrifying organisms corresponded with observed nitrification activities as indicated by the changes in measured nitrogen species concentrations. Furthermore, the coverage of the highly abundant MAGs (specifically, *Nitrosomonas*, *Sphingomonas*, and *Nitrospira*) corresponds with a previous report by Sakcham et al. (42), where, after removal of extracellular DNA (eDNA) from chloraminated drinking water, *Nitrosomonas* was present at a higher level of coverage than *Nitrospira.* Further, at locations with high nitrite concentrations and after eDNA removal, *Sphingomonas* showed an increase in relative abundance, which correlated with higher abundances of *Nitrosomonas* at this location. Bal Krishna et al. (43) observed an increase in abundance of *Sphingomonas* at lower chloramine concentrations and prior to the onset of nitrification. This was also observed in the present study. Here, the increase in abundance of *Sphingomonas* was associated with a reduction in disinfection residuals, which can result in the potential increase in heterotrophic bacterial growth and biofilm accumulation. However, the positive correlation between the *Sphingomonas* and *Nitrosomonas-*like MAGs, together with increased nitrite and nitrate concentrations, suggests that *Sphingomonas* may also act as an indicator for the onset of nitrification in chloraminated systems (42, 43).

### Nitrification was driven by cooccurring *Nitrosomonas* and *Nitrospira* species.

Nitrification in chloraminated drinking water systems has been extensively characterized (7, 8, 25). There is a significant body of reports of work that had used either culture-based or gene-targeted methods (e.g., amplicon sequencing, clone libraries, and quantitative PCR (qPCR) to investigate nitrification and nitrifying communities in drinking water systems at times when untargeted approaches (e.g., metagenomics) were largely unavailable or limited in use. The application of PCR-based approaches in earlier studies highlighted *Nitrosomonas* and *Nitrospira* species as the major ammonia and nitrite oxidizers, respectively, and provided descriptions of their role in nitrification in chloraminated drinking water systems (7, 8, 15, 44, 45). In this study, we use metagenomics to confirm the role of *Nitrosomonas* and *Nitrospira* species in nitrification. These nitrifiers consistently exhibited (i) high abundance at the SSU rRNA gene level, (ii) high coverage of nitrifying genes associated with *Nitrosomonas* (*amoCAB* and *hao* genes) and *Nitrospira* (*nxrAB* genes), and (iii) high coverage of *de novo*-reconstructed *Nitrosomonas-*like and *Nitrospira-*like MAGs across both reservoirs.

Here, the spatial changes in the concentrations of ammonium, nitrite, and nitrate and the high abundance of *Nitrosomonas* and *Nitrospira* strongly suggest that nitrogen-related activity (specifically nitrification) occurred within both chloraminated reservoirs. Duff et al. (46) reported that abundances of *amoA* genes from AOB in marine inertial bays showed significant correlations with the potential nitrification rate (PNR). The high abundance of genes involved in ammonia oxidation correlates with the observed increase in nitrate concentrations. It is also likely that the high abundance of *amo* genes could also have been a result of the presence of multiple gene copies within genomes of nitrifiers. AOBs have been found to contain two to three copies of *amoA* and *amoB* and multiple (including lone) copies of *amoC* genes (47, 48). However, this multiple-gene-copy issue is likely to also be present for other abundant nitrogen-transforming genes. Multiple gene copies have been also observed for *hao*, *nxrA*, and *nxrB* genes (49). Further, the presence of multiple gene copies in metagenomic data sets might not only have been a result of multiple copies within population genomes but might also have emerged from split genes due to breaks in contigs during the assembly process. Therefore, correcting for copy number for each nitrogen biotransformation gene may require extensive assumptions, in terms of both assembly breaks and copy numbers corresponding with taxonomic affiliation. However, considering that the spatial-temporal trends in abundance of nitrogen biotransformation genes correspond well with observed abundances of MAGs (less likely to be affected by both issues), this suggests that observations of gene abundance at the metagenome level do indeed correspond with relative abundances of the organisms that they originated from.

The phylogeny of the *amoA* gene confirmed that the majority of AOB were identified as strict ammonia oxidizers (specifically *Nitrosomonas* spp.) with the exception of one potential comammox *amoA* gene distinct from those of the canonical AOBs. Similarly, the phylogeny of *nxrA* genes showed that the majority of *nxrA* genes grouped within *Nitrospira* lineage II, which is the most widespread and diverse of the lineages, including canonical NOBs as well as all currently known comammox bacteria (12, 33, 50). The majority of *nxrA* genes identified in the present study grouped closely with those identified in canonical N. lenta and “*Ca.* Nitrospira sp. ST-bin5” bacteria (lacking *amo* and *hao* genes) (25). It is now known that both comammox and canonical AOB use ammonia as a substrate and can coexist in niches with low ammonia concentrations such as drinking water (25). However, other than the presence of a potential comammox *amoA* gene, no additional evidence was found that indicated the presence of comammox in this study, as *amo* and *hao* genes were not observed in the *Nitrospira*-like MAGs. It is plausible that whereas comammox bacteria are present in the two reservoirs, their low abundance resulted in highly fragmented contigs originating from their genomes and thus did not result in binning.

Typically, nitrification occurs in a modular fashion and is performed through cooperative and competitive interactions between ammonia and nitrite oxidizers (51, 52). Competitive interactions may exist within the groups of ammonia and nitrite oxidizers, which potentially compete for the substrates ammonia and nitrite, respectively (52). Strains within the N. oligotropha lineage have been shown to be adapted to very low to moderate ammonia concentrations and show increased affinities for ammonia compared to other *Nitrosomonas* species (26, 53). The use of a terminal restriction fragment length polymorphism (T-RFLP) and cloning approach also showed that some AOB representatives, specifically N. oligotropha, dominated chloraminated water and had a higher affinity for ammonia (7). Given the low concentrations of ammonia in these systems, the high affinity of N. oligotropha allows it to outcompete other *Nitrosomonas* species (7). This is likely to be the case in RES2, where ammonium concentrations are lower than in RES1. Here, where the *Nitrosomonas-*like MAG (C107) decreased in coverage, the other *Nitrosomonas-*like MAG (C58) increased in coverage, suggesting that C58 may have a higher affinity for ammonia and might therefore be able to outcompete other *Nitrosomonas*-like populations. Similarly, the *Nitrospira-*like MAG (C51) was more abundant than the *Nitrospira-*like MAG (C56), indicating its ability to outcompete other *Nitrospira* members in a manner similar to what has been demonstrated in other engineered systems (54, 55). Competition between *Nitrospira* spp. and separation in ecological niches may result from physiological properties such as affinities for nitrite and other substrates, formate utilization, and relationships with AOB (55). Here, the dominant *Nitrospira-*like MAG (C51) showed a positive relationship with the *Nitrosomonas-*like MAG (C107), decreasing in coverage in RES2, where disinfectant residuals and (consequently) ammonium concentrations were lower. This relationship may involve the tight coupling of canonical ammonia oxidation and nitrite oxidation, thereby resulting in the low concentrations of nitrite observed in both reservoirs (32, 33).

### Genetic potential of the microbial community for nitrogen metabolism.

Taxonomic classification suggested that the majority of MAGs represent genomes that are not yet represented in public databases. However, despite the limitations in available drinking water metagenomes, the results determined in this study provide insight into the diversity of the microorganisms involved in nitrogen transformation in chloraminated drinking water. As a consequence of nitrification, concentrations of nitrate increase, thereby providing an alternative form of biologically available nitrogen. The increased availability of nitrate potentially promotes the growth of highly diverse assemblages of microorganisms. Nitrogen transformations in the environment are typically carried out by microbial communities, which recycle nitrogen more efficiently than single microorganisms (52).

**FIG 4:**
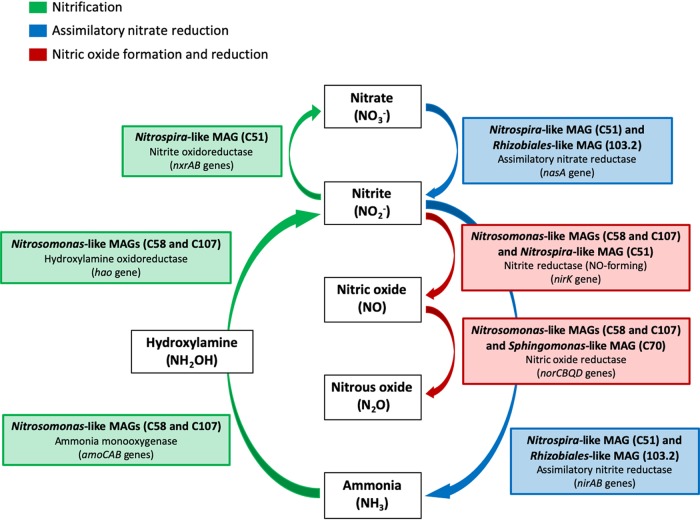
A schematic overview of the dominant genes and metagenome-assembled genomes (MAGs) involved in the major reactions of the nitrogen cycle in both chloraminated reservoirs. Reactions involved in nitrification are indicated in green, reactions involved in assimilatory nitrate reduction are indicated in blue, and reactions involving the formation and reduction of nitric oxide are indicated in red.

Where nitrification is driven by a select few microorganisms (i.e., *Nitrosomonas* and *Nitrospira* spp.), the reduction of nitrate, nitrite, and nitric oxide is likely performed by a diverse assemblage of bacteria (predominately *Alphaproteobacteria*, *Betaproteobacteriales*, and *Nitrospira*), each with its own discrete physiological requirements for optimal growth (52). The high abundance of *amoCAB* and *hao* (ammonia oxidation), *nxrAB* (nitrite oxidation), *nasA* (assimilatory nitrate reduction), *nirA* and *nirB* (nitrite reductase), *nirK* (nitrite reductase, NO formation), and *norCBQD* (nitric oxide reduction) genes suggests that nitrite and nitrate formed due to nitrification most likely are either (i) assimilatorily reduced to nitric oxide and (ultimately) nitrous oxide, potentially triggering biofilm formation, or (ii) fixed and converted to ammonia for assimilation when ammonia concentrations are low (Fig. 4).

Bacterial nitrate reduction has been shown to be a multifaceted process, performed by three distinct classes of nitrate-reducing systems differentiated by their cellular location, regulation, structure, chemical properties, and gene organization (56). In this study, all three systems (i.e., Nas, Nar, and Nap) were observed within the diverse group of recovered MAGs. The diversity observed within nitrate reductases in this study correlated with that observed in other studies where nitrate reductases were found to phylogenetically widespread (57–59). In many cases in this study, a single draft genome contained genes for both assimilatory and dissimilatory nitrate reduction processes. This indicates that these pathways may be interconnected and that the enzymes may play different roles under different metabolic conditions, facilitating adaptation to changes in nitrogen and/or oxygen conditions (56). The potential for assimilatory nitrate reduction was the dominant nitrate reduction pathway, as *nasA* genes were highly abundant and were the only nitrate reductase genes observed in the dominant MAGs, i.e., the *Nitrospira*-like MAG (C51) and the *Rhizobiales*-like MAG (C103.2). Assimilatory nitrate reductases are typically cytoplasmic and enable the utilization of nitrate as a nitrogen source. Expression of this enzyme is generally induced by nitrate but inhibited by ammonium; however, it is not affected by oxygen (56). We observed a converse relationship between the abundance of *nasA* genes and ammonia concentrations. The high abundance of genes associated with assimilatory nitrate reduction in this study correlated with reduced concentrations of ammonium and increased concentrations of nitrate. In the oligotrophic environment of DWDS, where nutrients are limited in availability, utilization of nitrate as a nitrogen source for biomass synthesis may be an important mechanism for survival (60).

The fate of nitrite formed through the reduction of nitrate may involve subsequent reduction to ammonia (through assimilatory nitrite reduction) or to nitric oxide (33, 56, 59). The positive correlation with the *nasA* and *nirA* genes suggests the potential for complete assimilatory nitrate reduction to ammonia, specifically in RES2. However, a converse relationship was observed between *nasA* and *nirA* genes in RES1, suggesting that *nirB* may potentially may play a larger role in the reduction of nitrite in RES1. The NirB nitrite reductase uses NADH as an electron donor to reduce nitrite in the cytoplasm, and high nitrite concentrations are typically needed for *nirB*, which is consistent with the proposed role of the NirB enzyme in detoxification of nitrite. This may be the case following RES1, where nitrite concentrations were generally higher than in RES2. Both dissimilatory nitrate reduction to ammonia (DNRA) and denitrification compete for the available nitrate and nitrite as an electron acceptor (61). It has been shown that DNRA may be favored when nitrate concentrations are low and organic electron donor availability is high, whereas denitrification outcompetes DNRA when nitrate concentrations are high and carbon supplies are limiting (60, 61). However, denitrification typically occurs in environments where oxygen availability is limited and nitrate is used in respiration. Therefore, denitrification may not play a significant role in an aerobic drinking water environment. Alternatively, nitrite may be reduced to nitric oxide (NO) via *nirK* (nitrite reductase, NO forming). At low, nontoxic concentrations, NO can potentially elicit cellular responses other than denitrification (62). Although it is difficult to separate NO signaling responses from detoxification and denitrification, it was previously observed in N. europaea that low concentrations of NO caused biofilm dispersal, whereas high concentrations of NO caused increased biofilm formation as a defense mechanism (62). *Sphingomonas* spp. have been reported to display conditional dimorphism between planktonic and sessile behavior depending on environmental conditions (30). In addition, *Nitrosomonas* spp. have been observed to exist in biofilms or suspended particle matter, specifically when ammonia concentrations are low (26, 63). The observed positive correlation between the *Sphingomonas-*like MAG and the *Nitrosomonas-*like MAGs may also be linked to this association with biofilms. While more research is needed, the regulation of NO may be linked to the planktonic/sessile state of *Sphingomonas* and its potential to initiate biofilm formation. In this study, a converse relationship was generally observed between assimilatory nitrite reduction (*nirA* and *nirB*) and NO-forming nitrite reduction (*nirK*), suggesting that when ammonia concentrations are higher, the need for nitrite to be assimilated to ammonia is potentially reduced and nitrite may be preferentially reduced to nitric oxide. Furthermore, *nor* genes are responsible for regulating the concentration of NO by the reduction of NO to nitrous oxide (N_2_O). As denitrification may not be an important process in this system, the observed high coverage of *nirK* and *nor* genes suggests that production of NO may result in its acting as an important molecule in regulation biofilm formation.

### Conclusion.

The complete transformation of nitrogen has been well characterized in many environments, including the open oceans and ocean sediments, wastewater, and agricultural soils. While nitrification has also been extensively studied in chloraminated drinking water systems, where nitrification is a major concern, to our knowledge, information is limited on the cooccurrence of nitrification potential (i.e., on the presence of genes and organism harboring those genes) with other metabolic traits that may affect nitrogen species availability via assimilatory or dissimilatory processes. Through the use of metagenomics, we confirmed previous findings indicating that *Nitrosomonas* and *Nitrospira* spp. are the main drivers of nitrification and, together with the water chemistry data (i.e., the data representing changes in ammonia, nitrite, and nitrate concentrations), this report improves our understanding nitrogen cycling potential in chloraminated drinking water. Furthermore, this approach has allowed us to (i) assemble the metagenomes of several members of the microbial community, (ii) analyze the phylogeny of genes responsible for nitrogen-transforming reactions, and (iii) investigate assimilatory genes for nitrate reduction that have not been the major focus of earlier research. Having used this approach, we propose that while canonical AOB and NOB drive nitrification in these chloraminated reservoirs, the nitrate formed during nitrification may be reduced (i) to ammonia by assimilatory processes when ammonia concentrations are low or (ii) to nitric oxide for potential regulation of biofilm formation when ammonia concentrations are not limiting. This report therefore provides insights into the genetic network behind microbially mediated nitrogen metabolism in chloraminated drinking water systems and maps out the fate of nitrogen species from chemoautotrophic nitrifiers to other commonly found heterotrophic bacteria in drinking water systems.

## MATERIALS AND METHODS

### Site description and sample collection.

**FIG 5:**
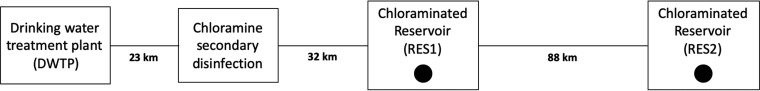
Simplified schematic showing the layout of the drinking water distribution system (DWDS) and the sample locations (reservoir 1 [RES1] and reservoir 2 [RES2] are indicated in the figure as black circles). Approximate distances between locations are indicated in the figure as dotted lines.

Sampling was conducted at two geographically separated but connected chloraminated reservoirs within a large South African DWDS, previously described by Potgieter et al. (24). Briefly, the process for treating surface water includes coagulation with polymeric coagulants, flocculation, sedimentation, pH adjustment with CO_2_ gas followed by filtration (rapid gravity sand filters), and, finally, initial disinfection with chlorine. Filter effluent is dosed with chlorine to achieve total residual chlorine concentrations between 1 and 1.5 mg/liter at the outlet of the drinking water treatment plant (DWTP). Chlorinated drinking water is then dosed with chloramine (0.8 to 1.5 mg/liter) at a secondary disinfection boosting station approximately 23 km from the DWTP. Here, monochloramine residuals range seasonally between 0.8 and 1.5 mg/liter within the chloraminated section of the DWDS. The first of the two reservoirs (RES1) sampled is located approximately 32 km from the secondary disinfectant boosting station. The second reservoir (RES2) is located approximately a further 88 km downstream from the first reservoir (Fig. 5). Samples were collected within a span of 2 years (October 2014 to September 2016) at the outlet of both reservoirs. Further details on a range of chemical parameters, including temperature, disinfectant residual concentrations (i.e., free chlorine, total chlorine, and monochloramine), and nitrogen species concentrations (i.e., ammonium, nitrite, and nitrate), were obtained from the utility (see Table S5 in the supplemental material).

10.1128/mSphere.00274-20.7 TABLE S5 Chemical monitoring data for the duration of the study obtained from the utility for both reservoirs. Download Table S5, DOCX file, 0.02 MB. Copyright © 2020 Potgieter et al. 2020 Potgieter et al. This content is distributed under the terms of the Creative Commons Attribution 4.0 International license.

### Sample processing.

Bulk water samples were collected in 8-liter sterile Nalgene polycarbonate bottles and transported to the laboratory on ice where they were kept at 4°C for 24 to 48 h until further processing. Samples were filtered to harvest microbial cells by pumping the collected bulk water through STERIVEX GP 0.22-μm-pore-size filter units (Millipore) using a Gilson Minipuls 3 peristaltic pump. The filters were kept in the dark and stored at –20°C until processing and DNA extraction were performed. A traditional phenol-chloroform extraction method optimized by Pinto et al. (64) and modified from Urakawa et al. (65) was used for the isolation of DNA from cells immobilized on filter membranes. Following extraction, 18 samples (i.e., 8 samples from RES1 and 10 samples from RES2) were selected for shotgun metagenomic sequencing based on their quality of DNA and temporal spread. The temporal spread of the samples is shown in Table S6.

10.1128/mSphere.00274-20.8 TABLE S6 Metagenome statistics, including sequencing depth and proportion of reads mapping to scaffolds greater than 500 bp in length, which were included in majority of the analyses. Download Table S6, DOCX file, 0.01 MB. Copyright © 2020 Potgieter et al. 2020 Potgieter et al. This content is distributed under the terms of the Creative Commons Attribution 4.0 International license.

### Metagenomic sequence processing, *de novo* assembly, functional annotation, and reference mapping.

Paired-end sequencing libraries were prepared using an Illumina TruSeq Nano DNA library preparation kit. Metagenomic sequencing was performed using the Illumina HiSeq 2500 sequence platform at the Agricultural Research Council—Biotechnology Platform (ARC-BTP), Gauteng, South Africa, resulting in 250-nucleotide (nt) paired-end reads (13,267,176 ± 3,534,751 reads per sample). Prior to assembly, the metagenomic reads were subject to adaptor removal and quality filtration using Trimmomatic (66) with a minimum sliding window quality score of 20 and reads shorter than 100 bp were discarded. Following quality filtering, the level of coverage of each metagenome was assessed using Nonpareil, a statistical program where read redundancy is used to estimate coverage (67). Prior to assembly, metagenomic reads were pooled and *de novo* assembly of quality trimmed reads into contiguous sequences (contigs) followed by scaffolding using metaSPAdes assembler version 3.9.0 (68) was performed with a kmers list consisting of 21, 33, 55, 77, 99, and 127. The resulting assembly consisted of 1,007,176 scaffolds (>500 bp) and an *N*_50_ and *L*_50_ of 1,638 bp and 42,230 bp, respectively. Reads consisting of more than 500 bp were mapped to the scaffolds, bam files were filtered to retain mapping reads (samtools view –F 4), and the number of reads mapping to the scaffolds in the metagenomics assembly was counted using an awk script. An average of 13,092,168 ± 3,561,160 reads per sample (>500 bp) were mapped to scaffolds (i.e., 99 ± 1.6% of reads mapped to scaffolds) (Table S6).

Open reading frames (ORFs) on scaffolds were predicted using Prodigal (69) with the meta flag activated. The resulting predicted ORFs were annotated against KEGG (Kyoto Encyclopedia of Genes and Genomes) (70) using DIAMOND (71). Genes involved in the nitrogen cycle were identified based on KEGG orthology (KO) numbers assigned to predict ORFs based on the KEGG nitrogen metabolism pathway. The abundance (reported as reads per kilobase per million [RPKM]) of genes was determined across all samples by dividing the number of reads mapping to the scaffold(s) containing the gene by the scaling factor (i.e., millions of reads per sample) and the length of the scaffold in kilobases.

### Metagenome-assembled genome (MAG) reconstruction.

Assembled scaffolds (>2,000 bp) from the coassembly of all samples were used to generate metagenomic assembled genomes (MAGs) using CONCOCT (72). This resulted in the construction of 115 CONCOCT clusters. A total of 60 CONCOCT clusters with completeness greater than 50%, based on the occurrence of 36 single-copy genes, used by CONCOCT to estimate completeness, were selected for further examination/refinement. The completeness and redundancy of these 60 CONCOCT clusters were checked with CheckM (73), with 47 clusters selected for further analysis based on 75% completeness. Of these, 3 had estimated redundancy levels greater than 10% and were manually refined using Anvi’o (74). This resulted in the identification of 47 medium-to-high-quality metagenome-assembled genomes (MAGs) (>70% complete, less than 10% redundancy). MAGs were functionally annotated and taxonomically classified using GhostKOALA (75), where predicted ORF’s were assigned KO numbers using KEGG’s set of nonredundant KEGG genes. The 47 constructed draft genomes were screened for key functional genes involved in KEGG nitrogen metabolism pathway. In addition, the abundance of all MAGs was calculated similar to that of the reference genomes (i.e., RPKM). MAGs were genetically compared to their closest related reference genome based on average amino acid identity (AAI) using MiGA (76). Taxonomic inference and characteristics of MAGs are detailed in Table S3. Genome-level inference of the 47 MAGs was then used to construct a phylogenomic tree using GToTree (77). Results of comparisons of differences in the levels of abundance (mapped reads) of MAGs between RES1 and RES2 were used to test for significant differences using DESeq2 (78) (Table S4).

### Marker gene-based taxonomic and phylogenetic analysis.

Small-subunit (SSU) rRNA gene sequences were identified using a hidden Markov model (HMM) search and the Infernal package (79) with domain-specific covariance models and were corrected as outlined previously (80). Detected SSU rRNA genes greater than 500 bp in length were classified using SILVA taxonomy, and the relative abundance of each SSU rRNA gene was estimated by dividing the total coverage of the scaffold containing the SSU rRNA gene by the coverage of all scaffolds containing SSU rRNA genes within each domain (i.e., bacteria, archaea, and eukaryota). Reference databases of ammonia monooxygenase subunit A (*amoA*; KO: K10944) and nitrite oxidoreductase subunit A (*nxrA*, KO: K00370) genes were created using corresponding reference sequences obtained from the NCBI GenBank Database with additional *nxrA* reference sequences obtained from Kitzinger et al. (81). An alignment was created for each gene using the MAFFT (version 7) online multiple alignment tool with the iterative refinement method L-INS-i (82). The resulting alignments were examined and trimmed by removing all overhangs, resulting in sequences that were equal in length. Aligned data sets were subjected to maximum likelihood analysis (83) in MEGA7 (Molecular Evolutionary Genetic Analysis) (84) using the best-fit substitution models as determined in MEGA7 model tests. For all maximum likelihood phylogenetic trees, branch support was estimated using nonparametric bootstrap analyses based on 1,000 pseudoreplicates determined under the same model parameters and rooted with appropriate outgroups. Amino acid sequences of genes annotated as *amoA* and *nxrA* were then placed on the respective reference phylogenetic trees using pplacer (85).

### Data accessibility.

All raw sequence data along with MAGs have been deposited in NCBI (BioProject accession number PRJNA524999). The MAGs are also available on figshare at the following URL: https://doi.org/10.6084/m9.figshare.12141960.
