# Supplementary figures and images for: Microbial Nitrogen Metabolism in Chloraminated Drinking Water Reservoirs
Source: mSphere. 2020 Apr 29;5(2):e00274-20. doi: 10.1128/mSphere.00274-20 (PMC7193043; doi:10.1128/mSphere.00274-20)

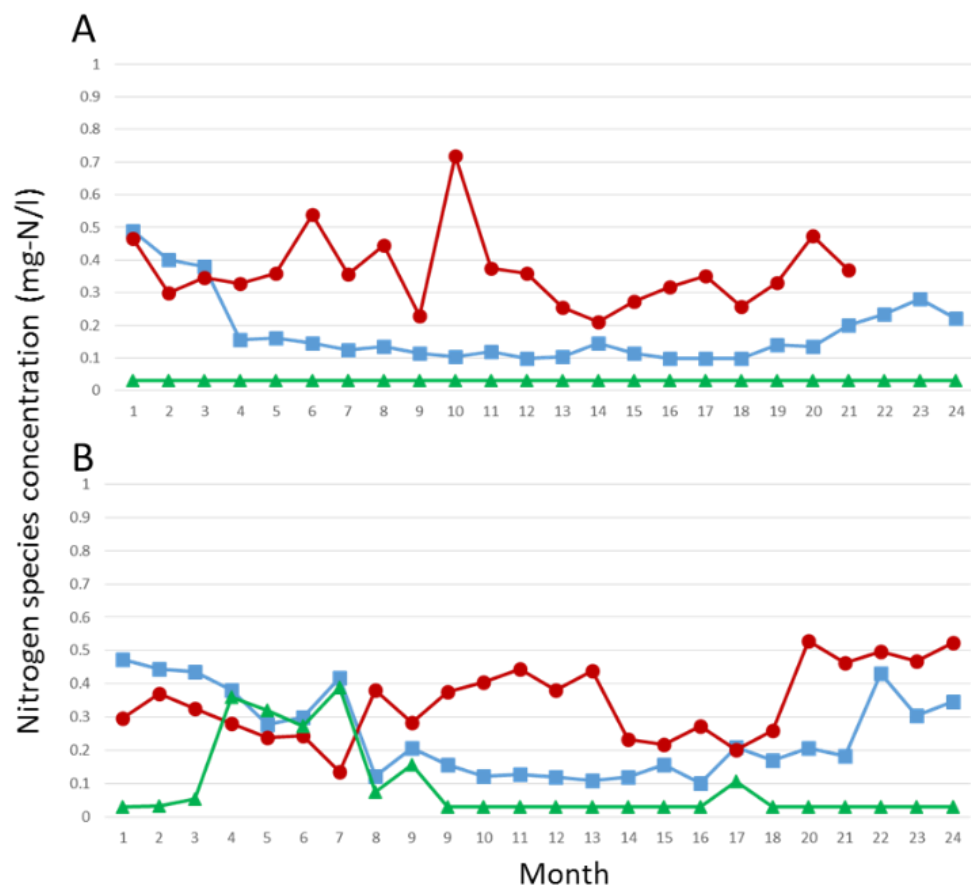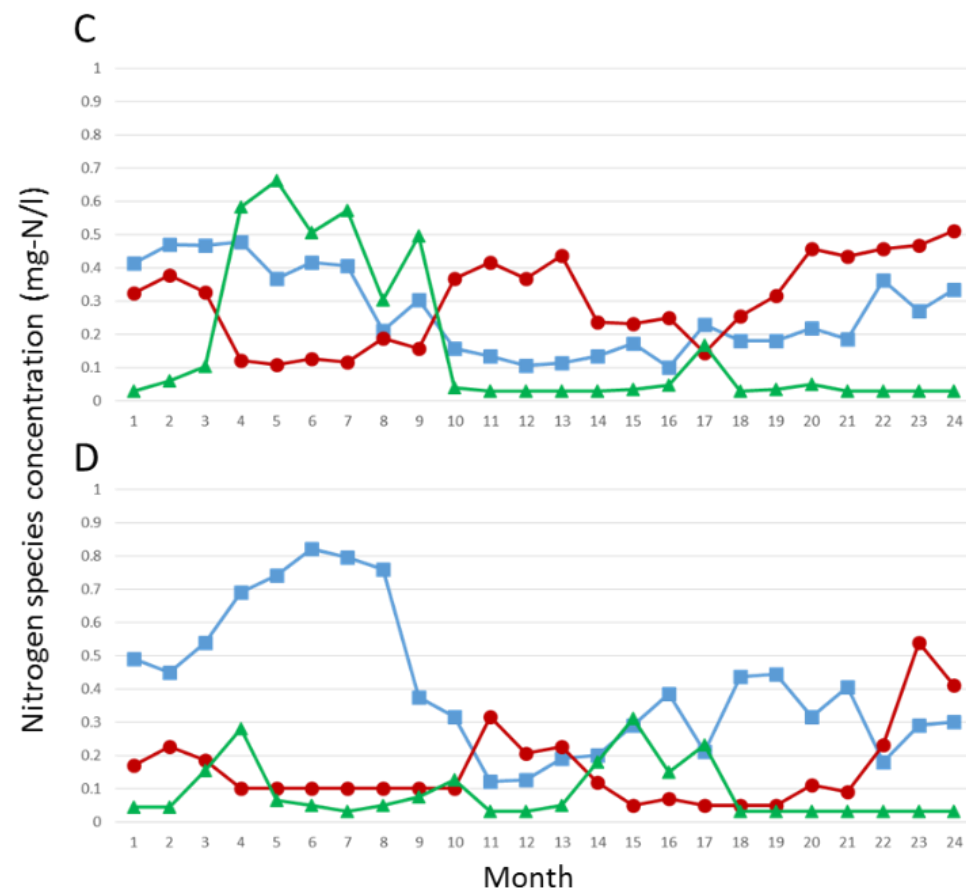

—●— Ammonium ( $\text{NH}_4^+$ )    —▲— Nitrite ( $\text{NO}_2^-$ )    —■— Nitrate ( $\text{NO}_3^-$ )

Supplement: FIG S1 [file mSphere.00274-20-sf001.pdf]

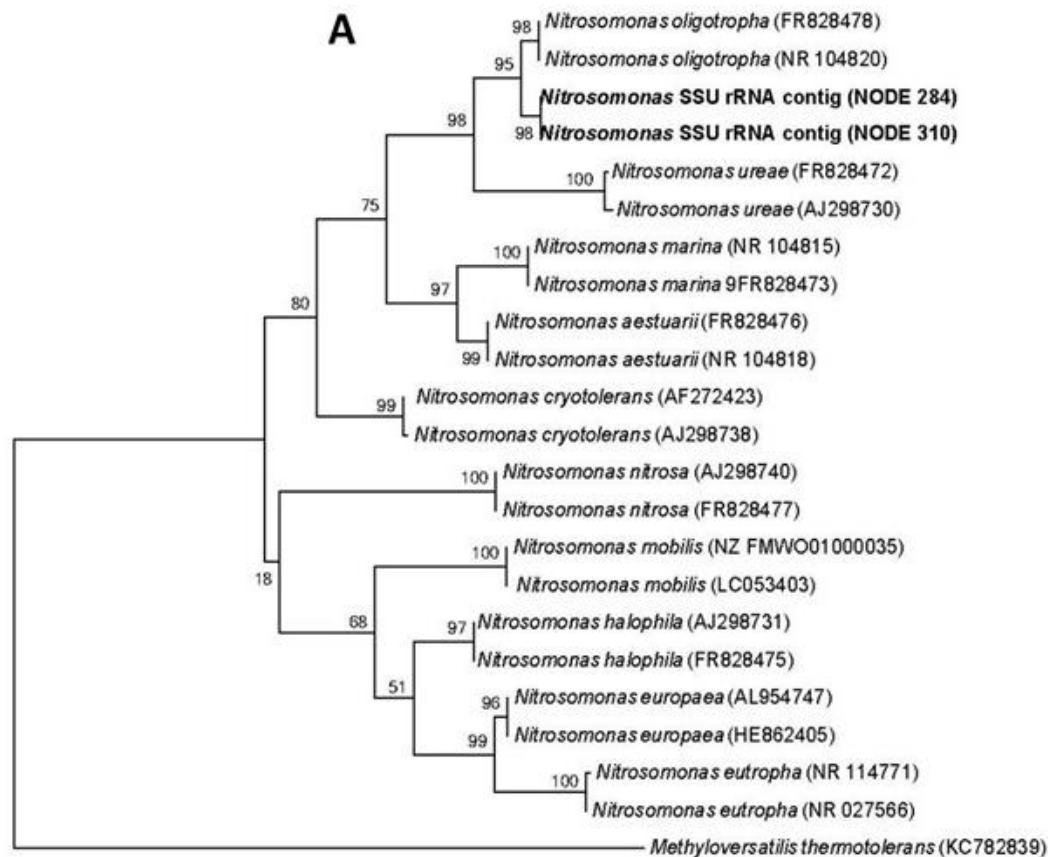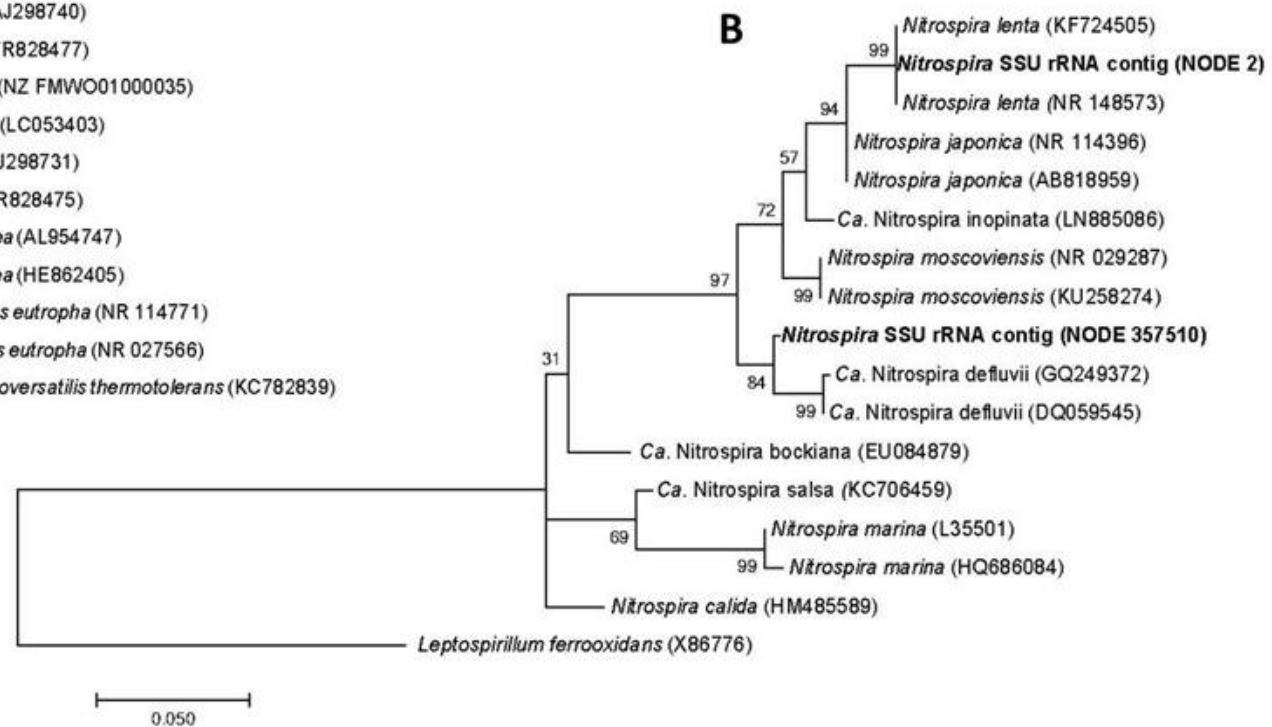

Supplement: FIG S2 [file mSphere.00274-20-sf002.pdf]
