# Supplementary material for: Microbial Nitrogen Metabolism in Chloraminated Drinking Water Reservoirs
Source: mSphere. 2020 Apr 29;5(2):e00274-20. doi: 10.1128/mSphere.00274-20 (PMC7193043; doi:10.1128/mSphere.00274-20)
Supplement: TABLE S1 [file mSphere.00274-20-st001.docx]

|  |  | | **RES1** | | **RES2** | |
| --- | --- | --- | --- | --- | --- | --- |
|  |  | | **MRA** | **SD** | **MRA** | **SD** |
| Bacterial phyla | *Proteobacteria* | | 81.60 | 13.32 | 82.98 | 10.28 |
|  |  | *Alphaproteobacteria* | 42.99 | 13.97 | 52.75 | 15.36 |
|  |  | *Deltaproteobacteria* | 0.08 | 0.08 | 0.09 | 0.09 |
|  |  | Total *Gammaproteobacteria* | 38.53 | 14.54 | 30.15 | 17.02 |
|  |  | *Gammaproteobacteria, Betaproteobacteriales* | 38.05 | 14.47 | 29.46 | 17.14 |
|  |  | Other *Gammaproteobacteria* | 0.48 | 0.37 | 0.69 | 0.35 |
|  | *Nitrospirota* | | 11.25 | 13.48 | 2.28 | 3.83 |
|  | *Bacteroidetes* | | 0.40 | 0.33 | 0.80 | 0.88 |
|  | *Planctomycetes* | | 0.16 | 0.14 | 0.58 | 0.94 |
|  | *Actinobacteria* | | 0.43 | 0.26 | 0.30 | 0.29 |
|  | *Acidobacteria* | | 0.12 | 0.12 | 0.27 | 0.28 |
|  | *Gemmatimonadetes* | | 0.19 | 0.18 | 0.10 | 0.16 |
|  | *Patescibacteria* | | 0.11 | 0.10 | 0.08 | 0.10 |
|  | *Verrucomicrobia* | | 0.04 | 0.07 | 0.10 | 0.10 |
|  | *Cyanobacteria* | | 0.02 | 0.03 | 0.09 | 0.11 |
|  | *Spirochaetes* | | 0.03 | 0.06 | 0.06 | 0.11 |
|  | *Chloroflexi* | | 0.03 | 0.07 | 0.05 | 0.06 |
|  | *Chlamydiae* | | 0.03 | 0.05 | 0.02 | 0.03 |
| Eukaryota |  | | 2.05 | 1.26 | 4.64 | 5.55 |
| Unclassified contigs |  | | 3.55 | 1.17 | 7.58 | 3.66 |

* MRA – Mean relative abundance

SD – Standard deviation
