# Supplementary material for: Microbial Nitrogen Metabolism in Chloraminated Drinking Water Reservoirs
Source: mSphere. 2020 Apr 29;5(2):e00274-20. doi: 10.1128/mSphere.00274-20 (PMC7193043; doi:10.1128/mSphere.00274-20)
Supplement: TABLE S2 [file mSphere.00274-20-st002.docx]

|  | **RES1** | | | **RES2** | | |
| --- | --- | --- | --- | --- | --- | --- |
| **Gene** | **Cumulative abundance (rpkm)** | **M** | **SD** | **Cumulative abundance (rpkm)** | **M** | **SD** |
| *amoA* (K10944) | 1143.07 | 142.88 | 79.54 | 1671.50 | 167.15 | 162.77 |
| *amoB* (K10945) | 2922.21 | 365.28 | 206.25 | 2165.83 | 216.58 | 171.42 |
| *amoC* (K10946) | 4625.20 | 578.15 | 287.34 | 4807.07 | 480.71 | 457.77 |
| *hao* (K10535) | 2484.77 | 310.60 | 149.43 | 2167.55 | 216.75 | 172.49 |
| *nasA* (K00372) | 648.00 | 81.00 | 35.68 | 1018.71 | 101.87 | 35.67 |
| *nirA* (K00366) | 683.49 | 85.44 | 43.70 | 552.49 | 55.25 | 19.54 |
| *nirB* (K00362) | 673.75 | 84.22 | 33.38 | 1159.78 | 115.98 | 46.56 |
| *nirK* (K00368) | 1094.54 | 136.82 | 46.45 | 927.00 | 92.70 | 40.42 |
| *norB* (K04561) | 1363.59 | 170.45 | 57.19 | 971.78 | 97.18 | 60.00 |
| *norC* (K02305) | 1060.08 | 132.51 | 50.50 | 424.05 | 42.41 | 14.50 |
| *norD* (K02448) | 2063.54 | 257.94 | 83.83 | 1786.84 | 178.68 | 88.06 |
| norQ (K04748) | 1903.40 | 237.92 | 89.33 | 1387.90 | 138.79 | 53.11 |
| nxrA (K00370) | 346.72 | 43.34 | 44.74 | 183.14 | 18.31 | 16.36 |
| nxrB (K00371) | 335.18 | 41.90 | 45.46 | 135.21 | 13.52 | 16.36 |

M – Mean relative abundance

SD – Standard deviation
