# Supplementary material for: Microbial Nitrogen Metabolism in Chloraminated Drinking Water Reservoirs
Source: mSphere. 2020 Apr 29;5(2):e00274-20. doi: 10.1128/mSphere.00274-20 (PMC7193043; doi:10.1128/mSphere.00274-20)
Supplement: TABLE S3 [file mSphere.00274-20-st003.docx]

| **MAG** | **Taxonomy^b^** | **MiGA** | **Completeness**  **%** | **Contamination**  **%** | **Quality %** | **G+C content %** | **Number of contigs** | **Genetic size (Mb)** | **Largest contig (bp)** | **N50** | **Number of ORF’s** | **Number of genes annotated** | **Coverage RES1 (rpkm)^c^** | **Coverage RES2 (rpkm)^c^** |
| --- | --- | --- | --- | --- | --- | --- | --- | --- | --- | --- | --- | --- | --- | --- |
| c2 | *Bacteroidetes*  *Cytophagia*  *Cytophagale*  *Flammeovirgaceae* | *Marivirga*  *tractuosa*  (50.15% AAI) | 91.9 | 2.7 | 78.4 | 41.79 | 396 | 3.87 | 82 589 | 14 095 | 3747 | 1452 (38.8%) | 0.1 ± 0.07 | 0.4 ± 0.46 |
| c3 | *Alphaproteobacteria*  *Rhodospirillales*  *Acetobacteraceae* | *Roseomonas* sp.  (56.54% AAI) | 94.6 | 0.9 | 90.1 | 69.05 | 292 | 4.52 | 125 874 | 24 612 | 4612 | 2024 (43.9%) | 1.32 ± 2.46 | 1.35 ± 1.71 |
| c4 | *Alphaproteobacteria*  *Caulobacterales*  *Caulobacteraceae* | *Caulobacteraceae*  bacterium  (52.29% AAI) | 91 | 0 | 91 | 67.51 | 163 | 2.72 | 83 313 | 25 390 | 2817 | 1389 (49.3%) | 0.79 ± 1.16 | 1.63 ± 1.3 |
| c14 | *Gammaprotebacteria* | *Thiohalobacter*  *thiocyanaticu*s  (48.24% AAI) | 90.1 | 1.8 | 81.1 | 67.88 | 729 | 5.51 | 34 030 | 9 353 | 5556 | 2325 (41.8%) | 0.14 ± 0.1 | 0.56 ± 0.53 |
| c19 | *Alphaproteobacteria*  *Sphingomonadales*  *Erythrobacteraceae*  *Porphyrobacter* | *Porphyrobacter* sp.  CACIAM  (81.5 ± 14,4% AAI) | 94.6 | 0.9 | 90.1 | 64.06 | 58 | 3.41 | 476 516 | 264 474 | 3279 | 1586 (48.4%) | 0.05 ± 0.01 | 4.6 ± 7.23 |
| c24 | *Gammaprotebacteria Betaproteobacteriales* | *Rhizobacter*  *gummiphilus*  (62.69% AAI) | 95.5 | 1.8 | 86.5 | 64.87 | 64 | 3.46 | 232 861 | 99 668 | 3260 | 1832 (56.2%) | 0.09 ± 0.06 | 0.49 ± 0.53 |
| c26 | *Betaproteobacteria*  *Methylophilales*  *Methylophilaceae*  *Methylotenera* | *Methylotenera*  *mobilis*  (62.03% AAI) | 95.5 | 1.8 | 86.5 | 41.94 | 12 | 2.03 | 735 558 | 469 317 | 2016 | 1268 (62.9%) | 0.02 ± 0.01 | 0.58 ± 1.17 |
| c27 | *Alphaproteobacteria*  *Rhizobiales*  *Bradyrhizobiaceae*  *Bosea* | *Bosea* sp.  PAMC  (79.03 ± 15.87% AAI) | 95.5 | 0.9 | 91 | 66.75 | 117 | 4.81 | 204 440 | 72 057 | 4670 | 2376 (50.9%) | 0.06 ± 0.02 | 2.6 ± 3.8 |
| c31 | *Alphaproteobacteria*  *Rhizobiales*  *Beijerinckiaceae* | *Methylocella*  *silvestris*  (56.71% AAI) | 95.5 | 1.8 | 86.5 | 62.55 | 58 | 4.43 | 399 037 | 106 052 | 4266 | 1942 (45.5%) | 0.08 ± 0.06 | 0.78 ± 1.06 |
| c33 | *Alphaproteobacteria*  *Rhizobiales*  *Bradyrhizolbiaceae*  *Oligotropha* | *Oligotropha*  *carboxidovorans*  (83.31 ± 12.94% AAI) | 94.6 | 1.8 | 85.6 | 61.68 | 51 | 3.45 | 253 596 | 122 000 | 3301 | 1794 (54.3%) | 0.05 ± 0.03 | 0.55 ± 0.82 |
| c34 | *Gemmatimonadetes*  *Gemmatimonadales*  *Gemmatimonadaceae*  *Gemmatirosa* | *Gemmatimonas*  *phototrophica*  (53.28% AAI) | 73 | 1.8 | 64 | 67.83 | 537 | 2.74 | 26 761 | 5 888 | 2892 | 1226 (42.4%) | 0.16 ± 0.08 | 0.51 ± 0.47 |
| c35 | *Alphaproteobacteria*  *Rhizobiales*  *Hyphomicrobiaceae*  *Hyphomicrobium* | *Hyphomicrobium*  *denitrificans*  (87.12 ±14.44% AAI) | 88.3 | 1.8 | 79.3 | 58.89 | 431 | 4.00 | 85 476 | 13 110 | 4239 | 1835 (43.3%) | 2.3 ± 2.22 | 1.12 ± 1 |
| c38 | *Alphaproteobacteria*  *Sphingomonadales*  *Sphingomonadaceae*  *Novosphingobium* | *Novosphingobium aromaticivorans*  (66.34% AAI) | 93.7 | 0.9 | 89.2 | 68.83 | 64 | 4.07 | 251 248 | 119 692 | 3727 | 1770 (47.5%) | 0.07 ± 0.07 | 0.53 ± 0.54 |
| c39 | *Gammaprotebacteria Betaproteobacteriales Sulfuricellaceae*  *Sulfuricella* | *Sulfuricella*  *denitrificans*  (84.31 ± 17.04% AAI) | 94.6 | 3.6 | 76.6 | 58.61 | 92 | 3.57 | 180 696 | 66 989 | 3485 | 1851 (53.1%) | 0.07 ± 0.02 | 0.5 ± 1.02 |
| c41 | *Alphaproteobacteria*  *Sphingomonadales*  *Erythrobacteraceae*  *Porphyrobacter* | *Porphyrobacter* sp.  LM 6  (97.28 ± 7.93% ANI) | 93.7 | 1.8 | 84.7 | 64.72 | 33 | 3.04 | 397 646 | 156 172 | 2951 | 1471 (49.8%) | 0.17 ± 0.1 | 3.74 ± 8.4 |
| c43 | *Alphaproteobacteria*  *Rhodospirillales* | *Azospirillum*  *brasilense*  (49.02% AAI) | 93.7 | 2.7 | 80.2 | 66.19 | 611 | 5.89 | 51 990 | 13 561 | 6212 | 2683 (43.2%) | 0.5 ± 0.56 | 0.38 ± 0.38 |
| c47 | *Gammaprotebacteria Betaproteobacteriales Gallionellaceae*  *Sideroxydans* | *Sideroxydans*  *lithotrophicus*  (77.27 ± 16,96% AAI) | 73 | 2.7 | 59.5 | 55.31 | 455 | 2.29 | 26 046 | 5 807 | 2655 | 1512 (56.9%) | 0.3 ± 0.35 | 0.21 ± 0.21 |
| c48.1^a^ | *Alphaproteobacteria*  *Sphingomonadales*  *Sphingomonadaceae*  *Sphingopyxis* | *Sphingopyxis*  *terrae*  (63.28% AAI) | 94.6 | 1.8 | 85.6 | 63.12 | 29 | 2.95 | 433 016 | 176 469 | 2853 | 1429 (50.1%) | 0.06 ± 0.03 | 1.85 ± 3.71 |
| c48.2^a^ | *Alphaproteobacteria*  *Rhizobiales*  *Bradyrhizobiaceae* | *Bradyrhizobium*  *lablabi*  (64.55% AAI) | 94.6 | 0.9 | 90.1 | 62.2 | 16 | 4.57 | 902 226 | 479 955 | 4332 | 1996 (46.1%) | 0.04 ± 0.01 | 1.68 ± 2.22 |
| c49 | *Alphaproteobacteria*  *Rhizobiales* | *Rhizobium*  *tropici*  (45.25% AAI) | 87.4 | 0.9 | 82.9 | 69.43 | 978 | 4.80 | 38 978 | 5 604 | 5423 | 2260 (41.7%) | 0.23 ± 0.28 | 0.6 ± 0.68 |
| c51 | *Nitrospirota*  *Nitrospirales*  *Nitrospiraceae*  *Nitrospira* | *Nitrospira*  *moscoviensis*  (62.69% AAI) | 92.8 | 5.4 | 65.8 | 57.78 | 80 | 4.27 | 1 125 973 | 247 953 | 4354 | 1608 (36.9%) | 16.93 ± 20.05 | 4.36 ± 6.92 |
| c56 | *Nitrospirota*  *Nitrospirales*  *Nitrospiraceae*  *Nitrospira* | *Nitrospira*  *moscoviensis*  (62.1% AAI) | 77.5 | 1.8 | 68.5 | 57.14 | 443 | 3.69 | 44 036 | 11 519 | 4121 | 1498 (36.4%) | 0.43 ± 0.5 | 0.23 ± 0.17 |
| c58 | *Gammaprotebacteria Betaproteobacteriales Nitrosomonadaceae*  *Nitrosomonas* | *Nitrosomonas* sp.  Is79A3  (66.34% AAI) | 94.6 | 0.9 | 90.1 | 48.69 | 70 | 3.12 | 237 347 | 90 243 | 2951 | 1486 (50.4%) | 7.12 ± 10.39 | 22.12 ± 27.3 |
| c59 | *Alphaproteobacteria*  *Rhodobacterales*  *Rhodobacteraceae* | *Rhodobacter* sp.  CZR27  (62.45% AAI) | 93.7 | 1.8 | 84.7 | 66.06 | 359 | 4.33 | 103 413 | 17 991 | 4620 | 2192 (47.4%) | 0.14 ± 0.08 | 0.51 ± 0.85 |
| c60 | *Gammaprotebacteria Betaproteobacteriales Methylophilaceae*  *Methylotenera* | *Methylotenera*  *mobilis*  (77.35 ± 15,15% AAI) | 95.5 | 1.8 | 86.5 | 45.61 | 24 | 2.53 | 486 922 | 217 332 | 2401 | 1424 (59.3%) | 0.02 ± 0.01 | 2.44 ± 4.84 |
| c61 | *Gammaprotebacteria Betaproteobacteriales Gallionellaceae*  *Sideroxydans/*  *Gallionella* | *Sideroxydans*  *lithotrophicus*  (67.0% AAI) | 94.6 | 0.9 | 90.1 | 56.92 | 50 | 2.75 | 341 276 | 100 797 | 2814 | 1477 (52.5%) | 1.65 ± 1.82 | 0.09 ± 0.1 |
| c64 | *Alphaproteobacteria*  *Caulobacterales* | *Caulobacteraceae*  bacterium  (52.17% AAI) | 87.4 | 0 | 87.4 | 65.98 | 313 | 2.43 | 54 901 | 10 673 | 2708 | 1354 (50.0%) | 0.8 ± 0.87 | 0.27 ± 0.26 |
| c65 | *Alphaproteobacteria*  *Sphingomonadales*  *Sphingomonadaceae* | *Sphingomonas*  *panacis*  (61.83% AAI) | 93.7 | 0 | 93.7 | 61.44 | 33 | 3.23 | 571 943 | 183 848 | 3132 | 1541 (49.2%) | 0.99 ± 1.73 | 1.18 ± 2.5 |
| c69 | *Gammaprotebacteria Betaproteobacteriales*  *Nitrosomonadaceae* | *Nitrosospira*  *lacus*  (52.37% AAI) | 93.7 | 1.8 | 84.7 | 60.83 | 82 | 3.91 | 228 320 | 102 579 | 3874 | 1967 (50.8%) | 0.05 ± 0.03 | 2.75 ± 4.57 |
| c70 | *Alphaproteobacteria*  *Sphingomonadales*  *Sphingomonadaceae*  *Sphingomonas* | *Sphingomonas*  *panacis*  (61.38% AAI) | 94.6 | 3.6 | 76.6 | 63.51 | 44 | 3.14 | 382 616 | 247 649 | 3082 | 1600 (51.9%) | 10.74 ± 18.11 | 11.95 ± 17.62 |
| c72 | *Alphaproteobacteria*  *Sphingomonadales*  *Sphingomonadaceae*  *Sphingomonas* | *Sphingomonas*  *panacis*  (62.63% AAI) | 93.7 | 0 | 93.7 | 61.81 | 31 | 3.89 | 611 207 | 391 571 | 3818 | 1682 (44.1%) | 2.76 ± 3.5 | 2.84 ± 5.13 |
| c74 | *Alphaproteobacteria*  *Rhizobiales*  *Hyphomicrobiaceae* | *Hyphomicrobium*  *nitrativorans*  (54.07% AAI) | 92.8 | 0.9 | 88.3 | 65.43 | 103 | 5.94 | 294 341 | 93 246 | 5453 | 2251 (41.3%) | 0.33 ± 0.72 | 1.07 ± 1.41 |
| c76 | *Gammaprotebacteria Betaproteobacteriales Gallionellaceae*  *Gallionella* | *Gallionella*  *capsiferriformans*  (79.91 ± 16.11% AAI) | 92.8 | 0.9 | 88.3 | 53.15 | 130 | 3.13 | 121 681 | 37 955 | 3102 | 1586 (51.1%) | 0.05 ± 0.02 | 0.34 ± 0.69 |
| c77 | *Gammaprotebacteria Betaproteobacteriales Methylophilaceae*  *Methylotenera* | *Methylotenera*  *versatilis*  (64.03% AAI) | 95.5 | 2.7 | 82 | 42.38 | 11 | 2.36 | 828 080 | 282 428 | 2257 | 1324 (58.7%) | 0.03 ± 0.03 | 0.62 ± 1.76 |
| c83 | *Gammaprotebacteria Betaproteobacteriales Gallionellaceae*  *Sideroxydans/*  *Gallionella* | *Gallionella*  *capsiferriformans*  (62.29% AAI) | 95.5 | 0.9 | 91 | 56.01 | 132 | 3.01 | 195 785 | 37 811 | 2977 | 1581 (53.1%) | 0.05 ± 0.03 | 0.6 ± 1.5 |
| c86 | *Alphaproteobacteria*  *Rhizobiales* | *Rhizobium* sp.  N324  (47.54% AAI) | 81.1 | 5.4 | 54.1 | 63.81 | 669 | 3.19 | 45 151 | 5 479 | 3618 | 1658 (45.8%) | 0.2 ± 0.23 | 0.52 ± 0.6 |
| c90 | *Alphaproteobacteria*  *Rhizobiales*  *Hyphomicrobiaceae* | *Hyphomicrobium*  *nitrativorans*  (54.02% AAI) | 93.7 | 0.9 | 89.2 | 63.9 | 87 | 5.15 | 412 933 | 130 872 | 4854 | 2111 (43.5%) | 4.07 ± 2.22 | 2.77 ± 0.97 |
| c93 | *Alphaproteobacteria*  *Rhizobiales*  *Methylobacteriaceae*  *Methylobacterium* | *Methylobacterium* sp. C1  (65.19% AAI) | 94.6 | 3.6 | 76.6 | 66.32 | 231 | 5.46 | 316 841 | 51 541 | 5451 | 2106 (38.6%) | 1.93 ± 3.36 | 2.57 ± 4.3 |
| c94 | *Planctomycetes*  *Planctomycetia*  *Planctomycetales*  *Planctomycetaceae* | *Singulisphaera*  *acidiphila*  (41.76% AAI) | 91.9 | 2.7 | 78.4 | 46.18 | 124 | 4.80 | 525 508 | 101 160 | 3712 | 1329 (35.9%) | 0.12 ± 0.08 | 0.44 ± 1.02 |
| c97 | Unknown  *Planctomycetes*  *Phycisphaerae* | *Phycisphaera*  *mikurensis*  (40.47% AAI) | 89.2 | 0 | 89.2 | 65.03 | 76 | 3.60 | 258 711 | 82 719 | 2976 | 1133 (38.1%) | 0.03 ± 0.02 | 0.68 ± 0.92 |
| c102 | *Betaproteobacteria*  *Nitrosomonadales*  *Gallionellaceae*  *Sideroxydans/*  *Gallionella* | *Sideroxydans*  *lithotrophicus*  (66.35% AAI) | 93.7 | 1.8 | 84.7 | 55.38 | 116 | 2.63 | 140 207 | 39 153 | 2675 | 1520 (56.8%) | 0.16 ± 0.07 | 0.23 ± 0.42 |
| c103.1^a^ | *Alphaproteobacteria*  *Rhizobiales*  *Hyphomicrobiaceae*  *Hyphomicrobium* | *Hyphomicrobium*  *denitrificans*  (61.59% AAI) | 95.5 | 7.2 | 59.5 | 59.99 | 12 | 3.65 | 1 305 095 | 829 204 | 3405 | 1705 (50.1%) | 1.92 ± 1.4 | 2.01 ± 1.75 |
| c103.2^a^ | *Alphaproteobacteria*  *Rhizobiales* | *Chelatococcus* sp.  (51.55% AAI) | 94.6 | 0.9 | 90.1 | 60.73 | 67 | 3.28 | 248 172 | 102 576 | 3179 | 1765 (55.5%) | 24.54 ± 12.15 | 19.91 ± 10.14 |
| c104 | *Alphaproteobacteria*  *Sphingomonadales*  *Sphingomonadaceae* | *Sphingomonas*  *panacis*  (61.76% AAI) | 94.6 | 0.9 | 90.1 | 66.91 | 96 | 3.25 | 161 741 | 54 966 | 3263 | 1554 (47.6%) | 4.91 ± 6.11 | 3.59 ± 2.98 |
| c107 | *Betaproteobacteria*  *Nitrosomonadales*  *Nitrosomonadaceae*  *Nitrosomonas* | *Nitrosomonas* sp.  Is79A3  (66.17% AAI) | 93.7 | 4.5 | 71.2 | 48.3 | 163 | 3.66 | 208 136 | 64 726 | 3461 | 1687 (48.7%) | 42.54 ± 26.31 | 11.82 ± 6.23 |
| c109 | *Betaproteobacteria*  *Nitrosomonadales*  *Nitrosomonadaceae* | *Nitrosospira* lacus  (52.47% AAI) | 95.5 | 0.9 | 91 | 62.68 | 59 | 3.80 | 299 525 | 118 187 | 3636 | 1832 (50.4%) | 0.33 ± 0.27 | 1.78 ± 4.76 |
| c114 | *Betaproteobacteria*  *Burkholderiales*  *Comamonadaceae* | *Acidovorax* sp.  NA3  (65.46% AAI) | 91.9 | 2.7 | 78.4 | 63.89 | 497 | 3.99 | 114 432 | 12 659 | 4261 | 2171 (51.0%) | 0.14 ± 0.06 | 0.91 ± 2.04 |

^a^ Manually curated Metagenome Assembled Genomes (MAGs)

^b^ Taxon was assigned when at least 75% of the identified genes resulted in a concordant taxonomy.

^c^ Values calculated as the mean coverage of each MAG across all sample within each reservoir (Mean ± Standard deviation)
