# Supplementary material for: Microbial Nitrogen Metabolism in Chloraminated Drinking Water Reservoirs
Source: mSphere. 2020 Apr 29;5(2):e00274-20. doi: 10.1128/mSphere.00274-20 (PMC7193043; doi:10.1128/mSphere.00274-20)
Supplement: TABLE S4 [file mSphere.00274-20-st004.docx]

| **Metagenome Assembled Genome** | **baseMean** | **log2Fold**  **Change** | **lfcSE** | **stat** | **pvalue** | **padj** |
| --- | --- | --- | --- | --- | --- | --- |
| Gammaproteobacteria_Betaproteobacteriales_Gallionellaceae_Sideroxydans (c61) | 46109.97 | -5.801 | 0.686 | -8.462 | 0.0000 | 0.0000 |
| Alphaproteobacteria_Sphingomonadales_Sphingomonadaceae_Porphyrobacter (c19) | 76092.02 | 5.403 | 0.936 | 5.774 | 0.0000 | 0.0000 |
| Alphaproteobacteria_Rhizobiales_Bradyrhizobiaceae (c48.2) | 27007.53 | 3.887 | 0.729 | 5.334 | 0.0000 | 0.0000 |
| Gammaproteobacteria_Betaproteobacteriales (c69) | 50215.97 | 4.686 | 0.891 | 5.261 | 0.0000 | 0.0000 |
| Alphaproteobacteria_Rhizobiales_Bradyrhizobiaceae_Bosea (c27) | 32122.24 | 3.883 | 0.765 | 5.077 | 0.0000 | 0.0000 |
| Alphaproteobacteria_Caulobacterales (c64) | 53172.66 | -3.157 | 0.654 | -4.826 | 0.0000 | 0.0000 |
| Unknown (c97) | 12322.93 | 2.812 | 0.605 | 4.645 | 0.0000 | 0.0000 |
| Gammaproteobacteria_Betaproteobacteriales_Methylophilaceae_Methylotenera (c60) | 24522.08 | 4.394 | 0.972 | 4.520 | 0.0000 | 0.0000 |
| Nitrospirota_Nitrospirales_Nitrospiraceae_Nitrospira (c56) | 21478.63 | -2.207 | 0.530 | -4.161 | 0.0000 | 0.0002 |
| Gammaproteobacteria_Betaproteobacteriales_Nitrosomonadaceae_Nitrosomonas (c107) | 2079592.42 | -2.332 | 0.632 | -3.691 | 0.0002 | 0.0010 |
| Gammaproteobacteria_Betaproteobacteriales (c109) | 17387.92 | -1.680 | 0.459 | -3.661 | 0.0003 | 0.0011 |
| Nitrospirota_Nitrospirales_Nitrospiraceae_Nitrospira (c51) | 749096.34 | -3.240 | 0.945 | -3.429 | 0.0006 | 0.0024 |
| Alphaproteobacteria_Sphingomonadales_Sphingomonadaceae_Sphingopyxis (c48.1) | 8779.36 | 2.051 | 0.622 | 3.299 | 0.0010 | 0.0035 |
| Alphaproteobacteria_Rhizobiales_Hyphomicrobiaceae (c74) | 22926.68 | 2.201 | 0.672 | 3.275 | 0.0011 | 0.0035 |
| Alphaproteobacteria_Rhizobiales_Bradyrhizolbiaceae_Oligotropha (c33) | 11296.24 | 1.796 | 0.588 | 3.053 | 0.0023 | 0.0071 |
| Alphaproteobacteria (c43) | 26130.83 | -1.688 | 0.588 | -2.871 | 0.0041 | 0.0120 |
| Gammaproteobacteria_Betaproteobacteriales_Methylophilaceae_Methylotenera (c26) | 3578.43 | 1.458 | 0.519 | 2.808 | 0.0050 | 0.0138 |
| Alphaproteobacteria_Sphingomonadales_Sphingomonadaceae_Novosphingobium (c38) | 12160.51 | 1.573 | 0.572 | 2.750 | 0.0060 | 0.0156 |
| Alphaproteobacteria_Rhizobiales (c31) | 14265.54 | 1.812 | 0.694 | 2.612 | 0.0090 | 0.0223 |
| Gammaproteobacteria_Betaproteobacteriales_Gallionellaceae (c47) | 23233.46 | -1.652 | 0.639 | -2.586 | 0.0097 | 0.0228 |
| Alphaproteobacteria_Sphingomonadales_Sphingomonadaceae (c72) | 176242.31 | -2.103 | 0.886 | -2.373 | 0.0177 | 0.0395 |
| Alphaproteobacteria_Rhizobiales_Hyphomicrobiaceae_Hyphomicrobium (c35) | 106677.89 | -1.714 | 0.741 | -2.314 | 0.0207 | 0.0429 |
| Alphaproteobacteria_Sphingomonadales (c65) | 16620.43 | -1.523 | 0.664 | -2.292 | 0.0219 | 0.0429 |
| Gammaproteobacteria_Betaproteobacteriales_Sulfuricellaceae_Sulfuricella (c39) | 6823.25 | 1.107 | 0.481 | 2.298 | 0.0215 | 0.0429 |
| Gammaproteobacteria_Betaproteobacteriales_Gallionellaceae_Gallionella (c76) | 5282.59 | 1.045 | 0.486 | 2.150 | 0.0315 | 0.0593 |
| Alphaproteobacteria_Sphingomonadales_Sphingomonadaceae_Porphyrobacter (c41) | 28626.37 | 1.288 | 0.648 | 1.989 | 0.0468 | 0.0845 |
| Gammaproteobacteria_Betaproteobacteriales_Nitrosomonadaceae_Nitrosomonas (c58) | 1818929.84 | 1.934 | 1.047 | 1.847 | 0.0647 | 0.1126 |
| Alphaproteobacteria_Rhodobacterales_Rhodobacteraceae (c59) | 5897.41 | -0.621 | 0.367 | -1.690 | 0.0911 | 0.1520 |
| Gammaproteobacteria_Betaproteobacteriales_Gallionellaceae_Sideroxydans (c102) | 8893.30 | -0.911 | 0.543 | -1.676 | 0.0938 | 0.1520 |
| Alphaproteobacteria_Rhizobiales (c90) | 332059.46 | -1.121 | 0.677 | -1.654 | 0.0981 | 0.1537 |
| Gammaproteobacteria_Betaproteobacteriales(c24) | 7430.22 | 0.860 | 0.530 | 1.621 | 0.1050 | 0.1592 |
| Alphaproteobacteria_Rhizobiales (c3) | 64711.16 | -1.428 | 0.899 | -1.589 | 0.1121 | 0.1647 |
| Bacteroidetes (c2) | 9018.01 | 0.786 | 0.538 | 1.460 | 0.1443 | 0.2056 |
| Gammaproteobacteria (c14) | 16145.96 | 0.697 | 0.498 | 1.399 | 0.1617 | 0.2236 |
| Gammaproteobacteria_Betaproteobacteriales_Gallionellaceae_Gallionella (c83) | 3981.33 | 0.369 | 0.338 | 1.093 | 0.2745 | 0.3686 |
| Gammaproteobacteria_Betaproteobacteriales_Comamonadaceae (c114) | 8586.97 | -0.376 | 0.360 | -1.042 | 0.2972 | 0.3880 |
| Alphaproteobacteria_Rhizobiales (c103.2) | 1957044.84 | -0.592 | 0.664 | -0.891 | 0.3729 | 0.4737 |
| Alphaproteobacteria_Rhizobiales_Methylobacteriaceae_Methylobacterium (c93) | 108734.74 | 0.597 | 0.701 | 0.851 | 0.3947 | 0.4882 |
| Alphaproteobacteria_Sphingomonadales_Sphingomonadaceae (c104) | 379959.96 | -0.693 | 0.945 | -0.733 | 0.4634 | 0.5445 |
| Gemmatimonadetes_Gemmatimonadales_Gemmatimonadaceae_Gemmatirosa (c34) | 14082.39 | 0.297 | 0.396 | 0.750 | 0.4534 | 0.5445 |
| Alphaproteobacteria_Caulobacterales (c4) | 65213.89 | 0.458 | 0.865 | 0.529 | 0.5967 | 0.6840 |
| Alphaproteobacteria (c86) | 19876.19 | 0.282 | 0.582 | 0.485 | 0.6277 | 0.7024 |
| Alphaproteobacteria_Sphingomonadales_Sphingomonadaceae (c70) | 972369.47 | 0.324 | 0.965 | 0.336 | 0.7370 | 0.8056 |
| Planctomycetes_Planctomycetales (c94) | 9903.90 | -0.171 | 0.591 | -0.289 | 0.7727 | 0.8254 |
| Alphaproteobacteria_Rhizobiales (c49) | 9746.86 | -0.067 | 0.532 | -0.126 | 0.8996 | 0.9395 |
| Alphaproteobacteria_Rhizobiales_Hyphomicrobiaceae_Hyphomicrobium (c103.1) | 137909.15 | -0.057 | 0.847 | -0.068 | 0.9460 | 0.9666 |
| Gammaproteobacteria_Betaproteobacteriales_Methylophilaceae_Methylotenera (c77) | 2911.44 | -0.010 | 0.340 | -0.030 | 0.9763 | 0.9763 |
