# Supplementary material for: Microbial Nitrogen Metabolism in Chloraminated Drinking Water Reservoirs
Source: mSphere. 2020 Apr 29;5(2):e00274-20. doi: 10.1128/mSphere.00274-20 (PMC7193043; doi:10.1128/mSphere.00274-20)
Supplement: TABLE S5 [file mSphere.00274-20-st005.docx]

| **RESERVOIR 1** |  | **Month** | **Free and available chlorine (mg/l)** | **Total chorine (mg/l)** | **Monochloramine (mg/l)** | **Total residual Chorine (mg/l)** | **Temperature (°C)** | **∆ ammonium (mg/l)** | **∆ nitrite (mg/l)** | **∆  nitrate (mg/l)** |
| --- | --- | --- | --- | --- | --- | --- | --- | --- | --- | --- |
|  | 1 | 07-Oct-14 | 0.53 | 2.09 | 1.56 | 2.12 | 16.1 | -0.17 | 0 | -0.02 |
|  | 2 | 04-Nov-14 | 0.3 | 2.2 | 1.9 | 2.2 | 18.5 | 0.07 | 0 | 0.04 |
|  | 3 | 02-Dec-14 | 0.03 | 0.74 | 0.71 | 0.81 | 22.3 | -0.02 | 0.02 | 0.06 |
|  | 4 | 13-Jan-15 | 0.07 | 1.94 | 1.87 | 1.94 | 23.4 | -0.05 | 0.33 | 0.23 |
|  | 5 | 03-Feb-15 | 0.11 | 2.07 | 1.96 | 2.09 | 22.7 | -0.12 | 0.29 | 0.12 |
|  | 6 | 03-Mar-15 | 0.34 | 0.36 | 0.02 | 0.36 | 22.2 | -0.3 | 0.24 | 0.16 |
|  | 7 | 14-Apr-15 | 0 | 0.15 | 0.15 | 0.18 | 20.8 | -0.22 | 0.36 | 0.29 |
|  | 8 | 05-May-15 | 0.06 | 1.26 | 1.2 | 1.28 | 18.4 | -0.06 | 0.04 | -0.01 |
|  | 9 | 02-Jun-15 | 0.06 | 0.67 | 0.61 | 0.68 | 16.8 | 0.05 | 0.13 | 0.09 |
|  | 10 | 07-Jul-15 | 0.18 | 1.35 | 1.17 | 1.68 | 10.5 | -0.34 | 0 | 0.05 |
|  | 11 | 04-Aug-15 | 0.09 | 0.96 | 0.87 | 0.95 | 12 | 0.03 | 0 | 0 |
|  | 12 | 01-Sep-15 | 0.11 | 1.66 | 1.55 | 1.61 | 14.7 | 0.08 | 0 | 0.03 |
|  | 13 | 06-Oct-15 | 0.01 | 1.42 | 1.41 | 1.42 | 19 | 0.12 | 0 | 0.02 |
|  | 14 | 03-Nov-15 | 0.12 | 1.69 | 1.57 | 1.74 | 19.5 | 0.23 | 0 | -0.04 |
|  | 15 | 01-Dec-15 | 0.06 | 0.11 | 0.05 | 0.13 | 20.7 | -0.04 | 0 | 0.01 |
|  | 16 | 05-Jan-16 | 0.03 | 0.1 | 0.07 | 0.14 | 22.7 | -0.1 | 0 | 0.06 |
|  | 17 | 02-Feb-16 | 0 | 0.33 | 0.33 | 0.35 | 23.2 | -0.08 | 0 | 0 |
|  | 18 | 08-Mar-16 | 0.06 | 0.27 | 0.21 | 0.3 | 23.5 | -0.06 | 0.08 | 0.11 |
|  | 19 | 05-Apr-16 | 0.03 | 0.48 | 0.45 | 0.59 | 21.7 | -0.07 | 0 | 0.03 |
|  | 20 | 10-May-16 | 0.13 | 1.07 | 0.94 | 1.07 | 18.1 | 0.06 | 0 | 0.07 |
|  | 21 | 07-Jun-16 | 0.24 | 0.84 | 0.6 | 0.97 | 15.2 | 0.09 | 0 | -0.02 |
|  | 22 | 05-Jul-16 | 0.15 | 2.11 | 1.96 | 2.11 | 12.8 | - | 0 | 0.2 |
|  | 23 | 02-Aug-16 | 0.09 | 1.84 | 1.75 | 1.99 | 11.9 | - | 0 | 0.02 |
|  | 24 | 30-Aug-16 | 0.21 | 1.94 | 1.73 | 1.86 | 14.2 | - | 0 | 0.13 |

| **RESERVOIR 2** | 1 | 10-Oct-14 | 0.13 | 0.64 | 0.51 | 0.98 | 17.9 | -0.15 | 0.02 | 0.08 |
| --- | --- | --- | --- | --- | --- | --- | --- | --- | --- | --- |
|  | 2 | 07-Nov-14 | 0.85 | 1.11 | 0.26 | 1.13 | 20.6 | -0.15 | -0.02 | -0.02 |
|  | 3 | 01-Dec-14 | 0.04 | 1 | 0.96 | 1 | 19.3 | -0.14 | 0.05 | 0.07 |
|  | 4 | 16-Jan-15 | 0.1 | 0.17 | 0.07 | 0.18 | 23 | -0.02 | -0.3 | 0.21 |
|  | 5 | 06-Feb-15 | 0.04 | 0.06 | 0.02 | 0.09 | 22.8 | -0.01 | -0.6 | 0.37 |
|  | 6 | 06-Mar-15 | 0.02 | 0.06 | 0.04 | 0.08 | 22.4 | -0.03 | -0.46 | 0.4 |
|  | 7 | 17-Apr-15 | 0.02 | 0.05 | 0.03 | 0.07 | 21.2 | -0.02 | -0.54 | 0.39 |
|  | 8 | 08-May-15 | 0.03 | 0.04 | 0.01 | 0.05 | 20 | -0.09 | -0.25 | 0.55 |
|  | 9 | 05-Jun-15 | 0 | 0.02 | 0.02 | 0.03 | 16.4 | -0.06 | -0.42 | 0.07 |
|  | 10 | 10-Jul-15 | 0.04 | 0.62 | 0.58 | 0.65 | 13.5 | -0.27 | 0.09 | 0.16 |
|  | 11 | 07-Aug-15 | 0.2 | 1.25 | 1.05 | 1.23 | 12.9 | -0.1 | 0 | -0.01 |
|  | 12 | 04-Sep-15 | 0.05 | 1.44 | 1.39 | 1.47 | 14.2 | -0.16 | 0 | 0.02 |
|  | 13 | 09-Oct-15 | 0.11 | 0.82 | 0.71 | 1.21 | 18.7 | -0.21 | 0.02 | 0.08 |
|  | 14 | 06-Nov-15 | 0.11 | 1.28 | 1.17 | 1.35 | 20.1 | -0.12 | 0.15 | 0.07 |
|  | 15 | 04-Dec-15 | 0.12 | 1.07 | 0.95 | 1.13 | 20.5 | -0.18 | 0.28 | 0.12 |
|  | 16 | 08-Jan-16 | 0.12 | 0.42 | 0.3 | 0.42 | 24 | -0.18 | 0.1 | 0.29 |
|  | 17 | 05-Feb-16 | 0.11 | 0.19 | 0.08 | 0.23 | 23.4 | -0.1 | 0.06 | -0.02 |
|  | 18 | 11-Mar-16 | 0.15 | 0.48 | 0.33 | 0.51 | 23.4 | -0.21 | 0 | 0.26 |
|  | 19 | 08-Apr-16 | 0 | 0.5 | 0.5 | 0.51 | 21.6 | -0.27 | 0 | 0.27 |
|  | 20 | 13-May-16 | 0.16 | 0.48 | 0.32 | 0.49 | 18 | -0.35 | -0.02 | 0.1 |
|  | 21 | 10-Jun-16 | 0.07 | 1.55 | 1.48 | 1.57 | 15.9 | -0.34 | 0 | 0.22 |
|  | 22 | 08-Jul-16 | 0.05 | 1.78 | 1.73 | 1.75 | 13.7 | -0.23 | 0 | -0.18 |
|  | 23 | 05-Aug-16 | 0.12 | 1.75 | 1.63 | 1.86 | 12.3 | 0.07 | 0 | 0.02 |
|  | 24 | 02-Sep-16 | 0.1 | 1.74 | 1.64 | 1.69 | 14.9 | -0.1 | 0 | -0.03 |
