# Supplementary material for: Microbial Nitrogen Metabolism in Chloraminated Drinking Water Reservoirs
Source: mSphere. 2020 Apr 29;5(2):e00274-20. doi: 10.1128/mSphere.00274-20 (PMC7193043; doi:10.1128/mSphere.00274-20)
Supplement: TABLE S6 [file mSphere.00274-20-st006.docx]

|  | **Sample** | **Month** | **Number of reads** | **Mapped reads to scaffolds**  **(> 500 bp)** | **% Mapped reads** |
| --- | --- | --- | --- | --- | --- |
| **1** | RES1_4 | Jan-15 | 14 249 511 | 14 195 821 | 99.62 |
| **2** | RES1_5 | Feb-15 | 13 019 437 | 12 929 842 | 99.31 |
| **3** | RES1_6 | Mar-15 | 12 747 382 | 12 673 029 | 99.42 |
| **4** | RES1_17 | Feb-16 | 14 647 502 | 14 573 146 | 99.49 |
| **5** | RES1_18 | Mar-16 | 18 488 103 | 18 376 301 | 99.4 |
| **6** | RES1_19 | Apr-16 | 11 184 973 | 11 116 758 | 99.39 |
| **7** | RES1_20 | May-16 | 6 855 217 | 6 753 636 | 98.52 |
| **8** | RES1_21 | Jun-16 | 16 944 215 | 16 809 348 | 99.2 |
| **9** | RES2_1 | Oct-14 | 12 231 070 | 11 382 585 | 93.06 |
| **10** | RES2_5 | Feb-15 | 11 677 750 | 11 487 402 | 98.37 |
| **11** | RES2_6 | Mar-15 | 6 919 446 | 6 758 814 | 97.68 |
| **12** | RES2_7 | Apr-15 | 11 590 760 | 11 452 070 | 98.8 |
| **13** | RES2_8 | May-15 | 13 020 429 | 12 809 835 | 98.38 |
| **14** | RES2_10 | Jul-15 | 13 802 699 | 13 303 643 | 96.38 |
